# Effect of different axillary management strategies on survival in elderly patients (≥70 Years) with early-stage breast cancer and 1–2 positive lymph nodes, stratified by risk factors

**DOI:** 10.1371/journal.pone.0353331

**Published:** 2026-11-11

**Authors:** Jin Chen, Jiefang Dai, Peng Zhou, Yewei Yuan

**Affiliations:** 1 Breast and Thyroid Department, Sichuan Provincial Maternity and Child Health Care Hospital, Sichuan Provincial Women’s and Children’s Hospital, The Affiliated Women’s and children’s Hospital of Chengdu Medical College, Chengdu, China; 2 Ultrasound Department, Sichuan Provincial Maternity and Child Health Care Hospital, Sichuan Provincial Women’s and Children’s Hospital, The Affiliated Women’s and children’s Hospital of Chengdu Medical College, Chengdu, China; 3 Department of Breast Surgery, Affiliated Hospital of Southwest Medical University, Luzhou, China; Netaji Subhash Chandra Bose Medical College and Hospital, INDIA

## Abstract

**Objective:**

Elderly patients (≥70 years) with early-stage breast cancer and 1–2 positive nodes exhibit markedly heterogeneous responses to different axillary management strategies, necessitating individualized treatment approaches. Specifically, SLNB combined with radiotherapy should be prioritized for patients in the high- and intermediate-risk groups, whereas SLNB alone may be sufficient for those in the low-risk group to minimize postoperative morbidity.

**Methods:**

The median follow-up time was 45 months. Following PSM, the SLNB plus radiotherapy (RT) regimen conferred significantly superior OS and BCSS compared to other axillary treatment modalities in the overall cohort. Multivariate analysis identified the following as independent prognostic risk factors for BCSS: age > 76 years (HR = 2.047, 95% CI: 1.603–2.614, P < 0.001), grade II–III disease (HR = 2.060, 95% CI: 1.452–2.923, P < 0.001), having 2 positive axillary nodes (HR = 1.335, 95% CI: 1.079–1.651, P = 0.008), tumor size 1.4–5 cm (HR = 1.667, 95% CI: 1.337–2.079, P < 0.001), triple-negative subtype (HR = 2.289, 95% CI: 1.534–3.416, P < 0.001), SLNB without RT (HR = 1.873, 95% CI: 1.363–2.575, P < 0.001), and ALND without RT (HR = 1.771, 95% CI: 1.291–2.430, P < 0.001). In subgroup analyses for BCSS, the 5-year rates for the high-risk cohort were 91.3% (SLNB+RT), 76.6% (SLNB without RT), 91.5% (ALND+RT), and 84.5% (ALND without RT). For the intermediate-risk cohort, the corresponding rates were 96.9%, 92.3%, 94.9%, and 92.7%. Kaplan-Meier analyses confirmed that SLNB+RT provided significant survival benefits in both the high- and intermediate-risk groups. For the low-risk cohort, the 5-year BCSS rates were 96.3%, 95.2%, 96.5%, and 94.3%, with no statistically significant differences among the four modalities (P > 0.05). Regarding LRR, the low-risk group experienced no recurrences across all four treatment arms within 5 years. In the intermediate-risk group, the 5-year LRR rates were 6.67% (SLNB+RT), 33.33% (SLNB without RT), 7.69% (ALND+RT), and 20.00% (ALND without RT); in the high-risk group, the corresponding rates were 7.69%, 50.00%, 7.70%, and 42.86%.

**Results:**

We retrospectively analyzed data from two cohorts: 8,977 patients diagnosed between 2010 and 2019 from the Surveillance, Epidemiology, and End Results (SEER) database, and 106 patients diagnosed between 2018 and 2021 at the Affiliated Hospital of Southwest Medical University. Propensity score matching (PSM) only was applied to the SEER‑database cohort (n = 8 977), yielding 5,858 matched patients divided equally into a sentinel lymph node biopsy (SLNB) group (n = 2,929) and an ALND group (n = 2,929). Clinicopathological characteristics were compared using the chi-square test and t-test. Multivariate Cox regression analysis was employed to determine prognostic factors for overall survival (OS) and BCSS, while logistic regression was used to identify risk factors for 5-year locoregional recurrence (LRR). Subgroup survival analyses stratified by different risk factors were performed using Kaplan-Meier methods, with intergroup comparisons conducted via the log-rank test.

**Conclusion:**

This study aimed to identify prognostic risk factors for breast cancer-specific survival (BCSS) in elderly patients (aged ≥70 years) with early-stage breast cancer and 1–2 positive lymph nodes. Additionally, we sought to evaluate the feasibility of omitting axillary lymph node dissection (ALND) and postoperative radiotherapy in selected patients to inform clinical decision-making.

## Introduction

Breast cancer is the most common malignancy and the leading cause of cancer-related death among women worldwide [1]. With the accelerating pace of population aging, the proportion of elderly breast cancer patients has increased annually. Approximately one-third of patients are aged 70 years or older at diagnosis [2]. However, optimal treatment strategies for this population remain controversial due to the frequent presence of comorbidities, age-related physiological decline, and underrepresentation in clinical trials [3].The publication of landmark trials such as ACOSOG Z0011 and IBCSG 23−01 has driven a “de-escalation” trend in axillary management for breast cancer [4]. The Z0011 trial demonstrated that for patients with T1–2 breast cancer, 1–2 positive sentinel nodes, and undergoing breast-conserving surgery with whole-breast RT, omitting ALND did not compromise OS or disease-free survival. However, these studies predominantly enrolled younger patients, leaving the optimal axillary strategy for elderly patients (≥70 years) with 1–2 positive nodes unclear [5].The 2016 Society of Surgical Oncology (SSO) guidelines recommended that SLNB may be omitted in patients aged ≥70 years with hormone receptor-positive, clinically node-negative early-stage breast cancer [6]. Nevertheless, these recommendations primarily address node-negative patients; for those with established 1–2 nodal metastases, the necessity of ALND and the value of postoperative RT remain inadequately supported by evidence. Moreover, the benefits of axillary surgery and RT likely differ significantly across patient subgroups defined by distinct risk profiles.Real-world evidence from the SEER database, combined with propensity score matching (PSM) to reduce selection bias [7], offers an opportunity to address these knowledge gaps. Therefore, this study aimed to evaluate the impact of different axillary surgical approaches (SLNB vs. ALND) with or without RT on survival in elderly patients (≥70 years) with early-stage breast cancer and 1–2 positive lymph nodes. Through risk-stratified subgroup analyses, we sought to identify patient subsets that derive differential benefit from each strategy, thereby providing evidence-based guidance for individualized clinical decision-making.

## Materials and methods

### Study population

This retrospective study utilized data from 17 SEER registry centers, encompassing patient demographics, tumor characteristics, treatment details, and survival outcomes. SEER primary cohort: Data from the SEER database was officially accessed and extracted on 02/03/2023. Data were extracted using SEER*Stat software (version 8.4.0.1) for 8,977 female patients aged ≥70 years diagnosed with early-stage breast cancer and 1–2 positive lymph nodes between January 1, 2010, and December 31, 2019. Variables included marital status, age, race, radiotherapy and chemotherapy administration, number of axillary nodes examined and positive, time from diagnosis to treatment, tumor size, molecular subtype, cause of death, survival months, and vital status.

An additional independent Chinese descriptive case‑series comprised 106 female patients aged ≥70 years diagnosed with early-stage breast cancer between January 2018 and December 2021 at the Affiliated Hospital of Southwest Medical University. The electronic medical records were retrieved for research analysis on 12/04/2026. Data collected included age, grade, number of positive axillary nodes, tumor size, and LRR status. Histological types were classified according to the International Classification of Diseases for Oncology, Third Edition (ICD-O-3).This study has received an ethics statement from the Affiliated Hospital of Southwest Medical University, exempting informed consent. All minimal datasets required to replicate findings of this study are publicly available at Mendeley Data, https://doi.org/10.17632/55brtt8hw9.1. The Chinese single‑center de‑identified patient dataset is included within the Mendeley Data repository.

### Inclusion and exclusion criteria

Inclusion criteria were: (1) first primary diagnosis of unilateral, non-special-type invasive ductal carcinoma in female patients aged ≥70 years; (2) clinical stage T1–2 according to the 7th and 8th editions of the American Joint Committee on Cancer (AJCC) TNM staging system; (3) receipt of breast and axillary surgery with or without breast reconstruction; (4) postoperative confirmation of 1–2 positive lymph nodes; (5) at least one axillary lymph node removed; and (6) complete clinical data available.

Exclusion criteria were: (1) breast cancer as a second primary malignancy; (2) male patients; (3) incomplete clinical data; (4) receipt of neoadjuvant chemotherapy; and (5) contralateral prophylactic mastectomy.

### Variable definitions and grouping

Continuous variables—age and tumor size—were categorized using X-tile software to identify optimal cut-off points: age (≤76 years vs. > 76 years) and tumor size (≤1.4 cm vs. 1.4–5 cm), the tumour‑size cut‑off value of 1.4 cm was derived from a prior study focused on breast‑conserving‑surgery patients [8]. Molecular subtypes were classified based on hormone receptor (HR) and human epidermal growth factor receptor 2 (HER2) status: HR + /HER2 + , HR + /HER2 − , HR − /HER2 + , and HR − /HER2− (triple-negative).

Because the SEER database does not explicitly document axillary surgical procedure, we defined SLNB as removal of 1–5 lymph nodes and ALND as removal of ≥6 nodes, consistent with AJCC guidelines [9] and prior literature [10,11].

After PSM, 5,858 patients were stratified by independent prognostic risk factors for BCSS: age > 76 years, grade II–III, two positive axillary nodes, tumor size 1.4–5 cm, and triple-negative subtype. Patients were categorized into three risk groups: low-risk (0 factors; n = 606, 10.3%), intermediate-risk (1 factor; n = 2,580, 44.1%), and high-risk (2–5 factors; n = 2,672, 45.6%).

### Study endpoints

The primary endpoint was BCSS, defined as the interval from initial diagnosis to breast cancer-specific death or last follow-up (November 2021). Secondary endpoints included OS (interval from diagnosis to death from any cause or last follow-up) and 5-year LRR (recurrence within 60 months post-surgery).

### Statistical analysis

Statistical analyses were performed using SPSS version 26.0 and R software version 4.1.3. To control for confounding, PSM was applied using the “MatchIt” package in R, with 1:1 nearest-neighbor matching and a caliper of 0.1, including all clinicopathological variables except axillary surgery type. Baseline characteristics between SLNB and ALND groups were compared using chi-square tests and t-tests.

Kaplan–Meier methods estimated survival probabilities for each axillary treatment modality, with intergroup comparisons using log-rank tests. Multivariate Cox regression models, incorporating variables significant in univariate analysis and those of clinical relevance (after excluding collinear variables), identified independent prognostic factors for BCSS. Logistic regression determined factors associated with 5-year LRR. Finally, risk-stratified subgroup survival analyses were conducted. A two-sided *P* < 0.05 was considered statistically significant.

## Results

### Baseline Characteristics Before and After PSM

A total of 8,977 eligible patients were included. After PSM, 5,858 patients remained, with balanced baseline characteristics between groups.

Before PSM, significant differences existed between the SLNB and ALND groups in race, grade, T stage, breast surgery type, radiotherapy/chemotherapy administration, time to treatment, number of positive nodes, ER/PR/HER2 status, molecular subtype, tumor size, annual income, and residence (*P* < 0.005). After PSM, all baseline characteristics were well balanced between the 2,929 patients in each group (*P* > 0.05 for all comparisons; Table 1).

**Table 1:** Clinicopathological characteristics of SLNB and ALND groups before and after PSM.

Clinicopathological Characteristics	Before PSM				After PSM			
	SLNB group (*n*=5447)	ALND group (*n*=3530)	*χ^2^*/*t*	*P value*	SLNB group (*n*=2929)	ALND group (*n*=2929)	*χ^2^*/*t*	*P value*
Marital Status			0.394	0.530			0.231	0.631
Unmarried Status	457(8.4)	283(8.0)			230(7.9)	240(8.2)		
Married Status	4990(91.6)	3247(92.0)			2699(92.1)	2689(91.8)		
Age			0.374	0.541			0.242	0.623
70–76 years	4683(86.0)	3051(86.4)			2517(85.9)	2530(86.4)		
>76 years	764(14.0)	479(13.6)			412(14.1)	399(13.6)		
Race			29.635	**<0.001**			0.763	0.663
White	4665(85.6)	2871(81.3)			2436(83.2)	2411(76.3)		
Black	427(7.8)	355(10.1)			267(9.1)	285(9.7)		
Other	355(6.5)	304(8.6)			226(7.7)	233(8.0)		
Histological Grade			147.610	**<0.001**			3.914	0.141
Grade I	1212(22.3)	573(16.2)			489(16.7)	498(17.0)		
Grade II	2987(54.8)	1754(49.7)			1609(54.9)	1538(52.5)		
Grade III	1248(22.9)	1203(34.1)			831(28.4)	893(30.5)		
Tumor Location			0.309	0.578			1.509	0.219
Left	2730(50.1)	1748(49.5)			1502(51.3)	1455(49.7)		
Right	2717(49.9)	1776(50.5)			1427(48.7)	1474(50.3)		
T Stage			102.941	**<0.001**			0.000	1.000
T_1_	3093(56.8)	1618(45.8)			1421(48.5)	1421(48.5)		
T_2_	2354(43.2)	1912(54.2)			1508(51.5)	1508(51.5)		
Breast Surgery Type			845.578	**<0.001**			1.322	0.250
Breast-Conserving Surgery	3955(72.6)	1479(41.9)			1493(51.0)	1449(49.5)		
Unilateral Mastectomy	1492(27.4)	2051(58.1)			1436(49.0)	1480(50.5)		
Radiotherapy Status			447.766	**<0.001**			2.181	0.140
No Radiotherapy	1495(27.4)	1744(49.4)			1238(42.3)	1294(44.2)		
Radiotherapy	3952(72.6)	1786(50.6)			1691(57.7)	1635(55.8)		
Chemotherapy Status			143.737	**<0.001**			2.261	0.133
No Chemotherapy	3955(72.6)	2136(60.5)			1923(65.7)	1868(63.8)		
Chemotherapy	1492(27.4)	1394(39.5)			1006(34.3)	1061(36.2)		
Time from Diagnosis to Treatment (months)	1.17±0.98	1.09±0.99	3.690	**<0.001**	1.12±0.89	1.11±1.02	0.694	0.488
Number of Positive Axillary Lymph Nodes			433.389	**<0.001**			0.375	0.540
1 Node	4539(83.3)	2261(64.1)			2124(72.5)	2103(71.8)		
2 Nodes	908(16.7)	1269(35.9)			805(27.5)	766(28.2)		
ER Status			60.594	**<0.001**			0.968	0.325
Negative	374(6.9)	410(11.6)			265(9.0)	287(9.8)		
Positive	5073(91.3)	3120(88.4)			2664(91.0)	2642(90.2)		
PR Status			61.172	**<0.001**			0.555	0.456
Negative	915(16.8)	769(23.5)			587(20.0)	610(20.8)		
Positive	4532(83.2)	2701(76.5)			2342(80.0)	2319(79.2)		
HER2 Status			18.062	**<0.001**			0.639	0.424
Negative	4949(90.9)	3109(88.1)			2622(89.5)	2603(88.9)		
Positive	498(9.1)	421(11.9)			307(10.5)	326(11.1)		
Molecular Subtype			76.130	**<0.001**			1.832	0.608
HR+,HER2-	410(7.5)	314(8.9)			244(8.3)	249(8.5)		
HR+,HER2+	4700(86.3)	2768(80.1)			2442(83.4)	2414(76.4)		
HR-,HER2+	88(1.6)	107(3.0)			63(2.2)	77(2.6)		
HR-,HER2-	249(4.6)	281(8.0)			180(6.1)	189(6.5)		
Tumor Size			40.494	**<0.001**			0.055	0.815
≤1.4cm	4634(85.1)	2761(79.9)			2371(80.9)	2378(81.2)		
1.4cm-5cm	813(14.9)	709(20.1)			558(19.1)	551(18.8)		
Tumor Quadrant			9.363	0.095			1.939	0.858
Central Region	476(8.8)	363(10.3)			277(9.5)	301(10.3)		
Upper Inner Quadrant	520(9.5)	315(8.9)			279(9.5)	269(9.2)		
Lower Inner Quadrant	340(6.2)	203(5.8)			178(6.1)	168(5.7)		
Upper Outer Quadrant	2174(39.9)	1359(38.5)			1154(39.4)	1148(39.2)		
Lower Outer Quadrant	525(9.6)	329(9.3)			280(9.6)	267(9.1)		
Other Site	1406(25.8)	961(27.2)			761(26.0)	776(26.5)		
Annual Income (×10^3^, USD)			17.572	**<0.001**			1.002	0.606
<55	1256(23.1)	951(26.9)			701(23.9)	716(24.4)		
55~74	2418(44.4)	1475(41.8)			1314(44.9)	1276(43.6)		
≥75	1773(32.6)	1104(31.3)			914(31.2)	937(32.0)		
Residence			14.597	**0.006**			9.240	0.055
Counties under Urban Areas with Population >1 Million	3040(55.8)	1924(54.5)			1640(56.0)	1615(55.1)		
Counties under Urban Areas with Population 250,000–1 Million	1294(23.8)	835(23.7)			662(22.6)	676(23.1)		
Counties under Urban Areas with Population <250,000	441(8.1)	335(9.5)			238(8.1)	286(9.8)		
Non-Urban Counties Adjacent to Urban Areas	404(7.4)	222(6.3)			232(7.9)	189(6.5)		
Non-Urban Counties Not Adjacent to Urban Areas	268(4.9)	214(6.1)			157(5.4)	163(5.6)		

### Survival analysis for the overall cohort after PSM

With a median follow-up of 45 months, 5-year BCSS rates for the overall cohort were: SLNB+RT 94.4%, SLNB without RT 88.8%, ALND+RT 93.3%, and ALND without RT 89.4%. Corresponding 5-year OS rates were: SLNB+RT 86.9%, SLNB without RT 73.7%, ALND+RT 85.4%, and ALND without RT 73.9%. Kaplan–Meier analysis demonstrated that SLNB+RT conferred significantly superior OS and BCSS compared to all other treatment modalities (Fig 1, Fig 2, Table 2).

**Table 2:** Pairwise comparisons of OS and BCSS among axillary treatment modalities.

Impact of Different Axillary Management Modalities on OS in the Overall Cohort of Elderly Patients with Early-Stage Breast Cancer After PSM						
Axillary Treatment Modality	SLNB+RT (86.9%)		SLNB without RT (73.7%)		ALND+RT (85.4%)	
	*χ* ^*2*^	*P* value	*χ* ^*2*^	*P* value	*χ* ^*2*^	*P* value
SLNB without RT (73.7%)	45.928	**<0.001**	–	–	–	–
ALND+ RT (85.4%)	0.032	0.858	48.551	**<0.001**	–	–
ALND without RT (73.9%)	38.807	**<0.001**	0.295	0.587	43.171	**<0.001**
Impact of Different Axillary Management Modalities on BCSS in the Overall Cohort of Elderly Patients with Early-Stage Breast Cancer After PSM						
Axillary Treatment Modality	SLNB+ RT (94.4%)		SLNB without RT (88.8%)		ALND+ RT (93.3%)	
	*χ* ^*2*^	*P* value	*χ* ^*2*^	*P* value	*χ* ^*2*^	*P* value
SLNB without RT (88.8%)	17.633	**<0.001**	–	–	–	–
ALND+ RT (93.3%)	0.302	0.583	13.987	**<0.001**	–	–
ALND without RT (89.4%)	15.953	**<0.001**	0.060	0.807	12.687	**<0.001**

**Fig 1:**
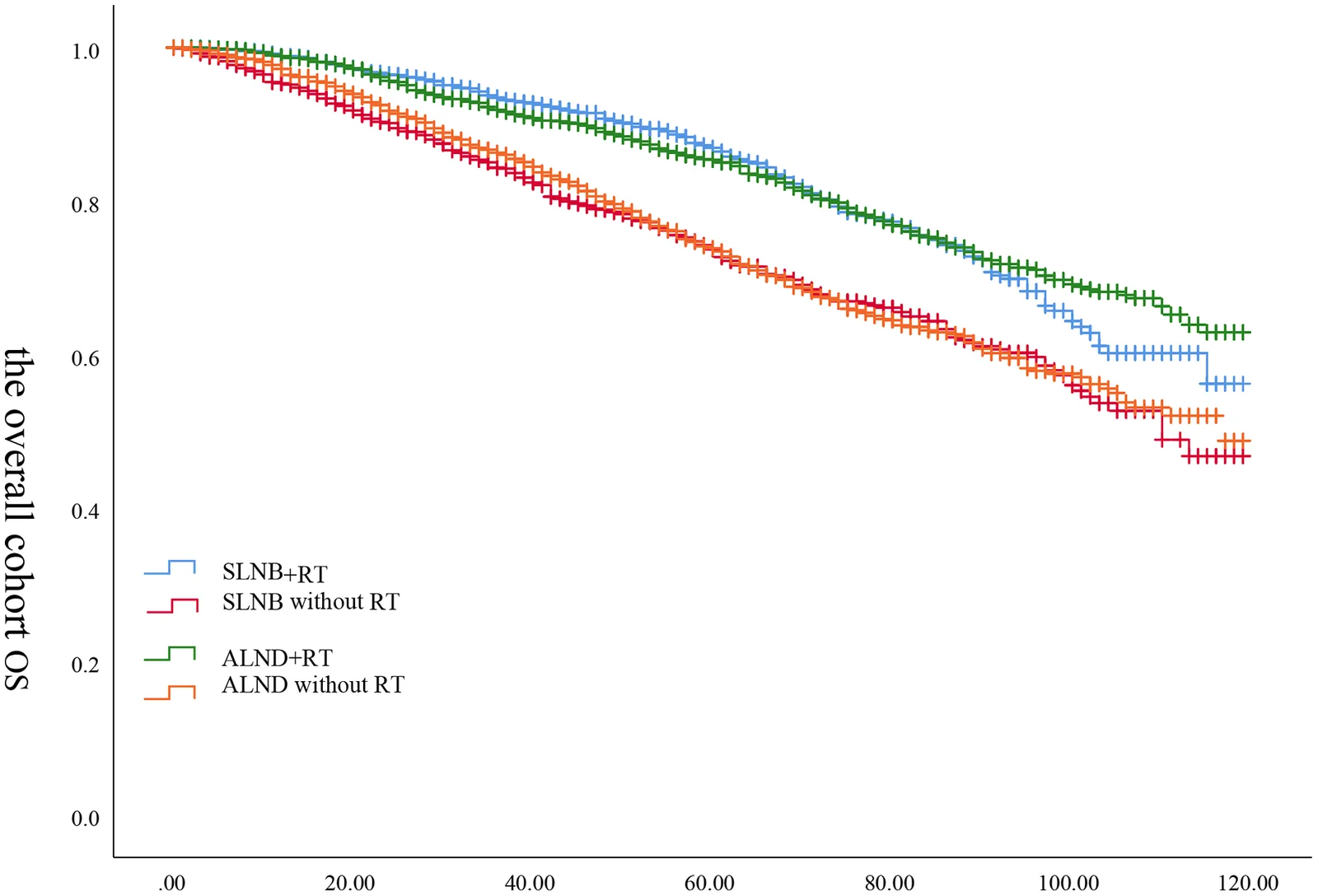
Kaplan–Meier curves for OS according to axillary treatment modality after PSM.

**Fig 2:**
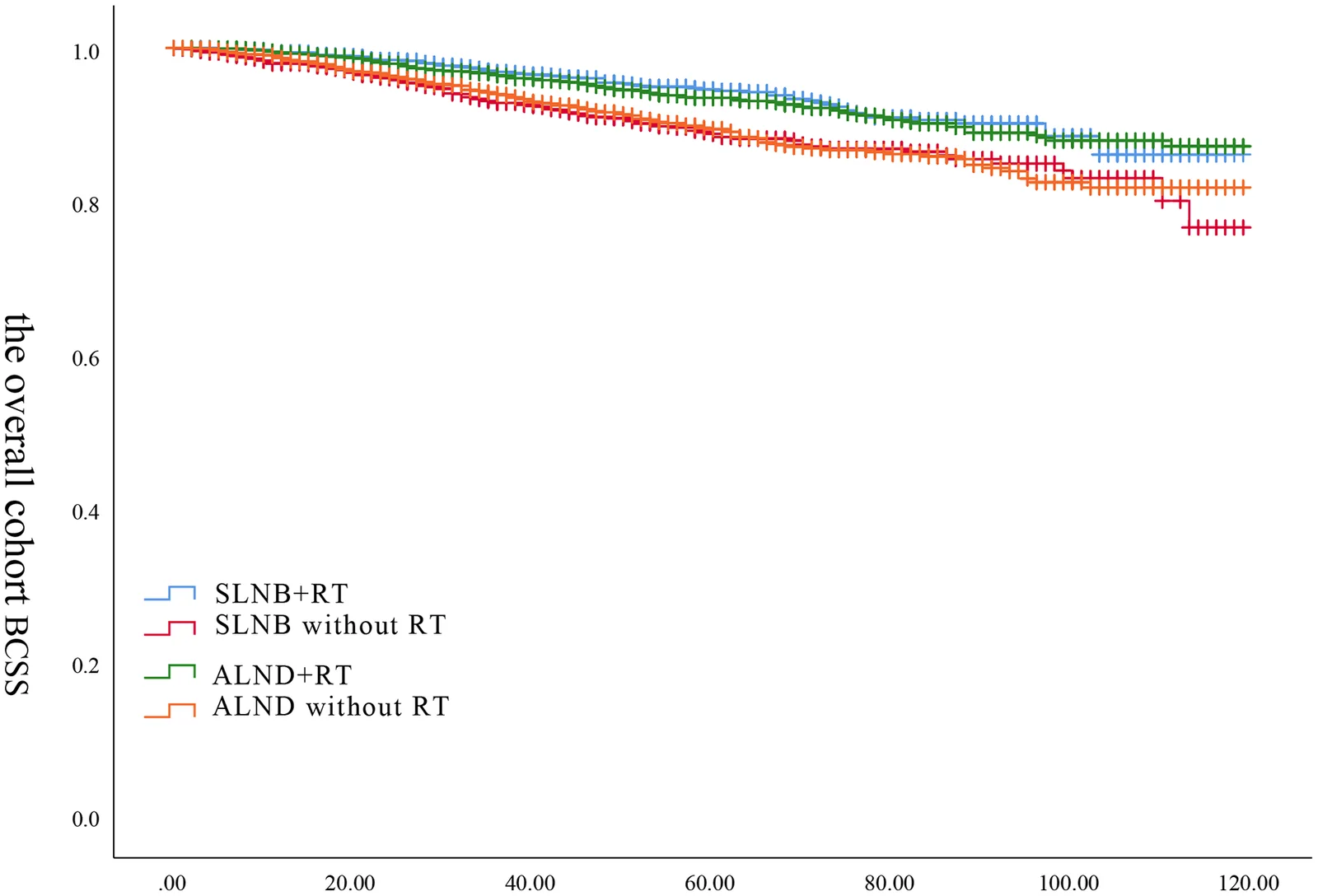
Kaplan–Meier curves for BCSS according to axillary treatment modality after PSM.

### Univariate and multivariate cox regression analysis for BCSS

Univariate analysis identified age, race, grade, T stage, breast surgery type, radiotherapy/chemotherapy, number of positive nodes, ER/PR status, molecular subtype, tumor size, annual income, and axillary treatment modality as significantly associated with BCSS (*P* < 0.05). Multivariate analysis retained age, grade, number of positive nodes, tumor size, molecular subtype, annual income, and axillary treatment modality as independent prognostic factors (Table 3, Table 4). Specifically, the following were independent adverse prognostic factors for BCSS: age > 76 years (HR = 2.047, 95% CI: 1.603–2.614, *P* < 0.001), grade II–III (HR = 2.060, 95% CI: 1.452–2.923, *P* < 0.001), two positive nodes (HR = 1.335, 95% CI: 1.079–1.651, *P* = 0.008), tumor size 1.4–5 cm (HR = 1.667, 95% CI: 1.337–2.079, *P* < 0.001), triple-negative subtype (HR = 2.289, 95% CI: 1.534–3.416, *P* < 0.001), SLNB without RT (HR = 1.873, 95% CI: 1.363–2.575, *P* < 0.001), and ALND without RT (HR = 1.771, 95% CI: 1.291–2.430, *P* < 0.001).

**Table 3:** Univariate Cox regression analysis for BCSS.

Clinicopathological Characteristics	Regression Coefficient	Standard Error (SE)	Wald*χ*^*2*^	*HR* Value	*HR* 95%*CI*	*P* Value
Marital Status						
Unmarried Status					1	
Married Status	0.268	0.199	1.818	1.308	(0.885,1.932)	0.178
Age						
70–76 years					1	
>76 years	0.846	0.116	52.755	2.331	(1.855,2.928)	**<0.001**
Race						
White					1	
Black	0.180	0.163	1.213	1.197	(0.869,1.650)	0.271
Other	−0.077	0.197	0.152	0.926	(0.630,1.361)	0.696
Histological Grade						
Grade I					1	
Grade II	0.465	0.185	6.347	1.592	(1.109,2.285)	**0.012**
Grade III	1.363	0.180	57.368	3.907	(2.746,5.560)	**<0.001**
Tumor Location						
Left					1	
Right	0.080	0.098	0.663	1.083	(0.893,1.314)	0.416
T Stage						
T_1_					1	
T_2_	0.711	0.104	46.943	2.036	(1.661,2.495)	**<0.001**
Breast Surgery Type						
Breast-Conserving Surgery					1	
Unilateral Mastectomy	0.298	0.099	8.983	1.347	(1.108,1.636)	**0.003**
Radiotherapy Status						
No Radiotherapy					1	
Radiotherapy	−0.539	0.099	29.448	0.583	(0.480,0.709)	**<0.001**
Chemotherapy Status						
No Chemotherapy					1	
Chemotherapy	0.210	0.099	4.483	1.234	(1.016,1.500)	**0.034**
Time from Diagnosis to Treatment (months)	0.011	0.051	0.042	1.011	(0.914,1.118)	0.838
Number of Positive Axillary Lymph Nodes						
1 Node					1	
2 Nodes	0.225	0.105	4.534	1.252	(1.018,1.539)	**0.033**
ER Status						
Negative					1	
Positive	−0.845	0.123	46.973	0.429	(0.337,0.547)	**<0.001**
PR Status						
Negative					1	
Positive	−0.870	0.101	73.498	0.419	(0.344,0.511)	**<0.001**
HER2 Status						
Negative					1	
Positive	0.161	0.147	1.203	1.175	(0.881,1.568)	0.273
Molecular Subtype						
HR + ,HER2-					1	
HR + ,HER2+	−0.276	0.167	2.871	0.754	(0.544,1.045)	0.090
HR-,HER2+	0.014	0.328	0.002	1.014	(0.533,1.929)	0.967
HR-,HER2-	0.761	0.199	14.572	2.141	(1.448,3.166)	**<0.001**
Tumor Size						
≤1.4 cm					1	
1.4 cm-5 cm	0.705	0.109	42.013	2.023	(1.635,2.504)	**<0.001**
Tumor Quadrant						
Central Region					1	
Upper Inner Quadrant	0.279	0.218	1.641	1.322	(0.862,2.028)	0.200
Lower Inner Quadrant	0.118	0.255	0.216	1.126	(0.684,1.854)	0.642
Upper Outer Quadrant	−0.011	0.176	0.004	0.989	(0.693,1.411)	0.950
Lower Outer Quadrant	0.023	0.233	0.010	1.023	(0.648,1.614)	0.922
Other Site	0.122	0.187	0.425	1.130	(0.783,1.629)	1.130
Annual Income (×10^3^, USD)						
<55					1	
55 ~ 74	−0.008	0.116	0.005	0.992	(0.790,1.245)	0.943
≥75	−0.371	0.137	7.311	0.690	(0.527,0.903)	**0.007**
Residence						
Counties under Urban Areas with Population >1 Million					1	
Counties under Urban Areas with Population 250,000–1 Million	−0.151	0.128	1.380	0.860	(0.669,1.106)	0.240
Counties under Urban Areas with Population <250,000	0.272	0.160	2.874	1.312	(0.958,1.796)	0.090
Non-Urban Counties Adjacent to Urban Areas	0.096	0.200	0.232	1.101	(0.744,1.631)	0.630
Non-Urban Counties Not Adjacent to Urban Areas	0.254	0.200	1.612	1.290	(0.871,1.910)	0.204
Axillary Treatment Modality						
SLNB+RT					1	
SLNB without RT	0.621	0.150	17.199	1.861	(1.388,2.496)	**<0.001**
ALND+ RT	0.107	0.152	0.498	1.113	(0.766,1.500)	0.480
ALND without RT	0.581	0.146	15.842	1.788	(1.343,2.380)	**<0.001**

**Table 4:** Multivariate Cox regression analysis for BCSS.

Clinicopathological Characteristics	Regression Coefficient	Standard Error (SE)	Wald*χ*^*2*^	*HR* Value	*HR* 95%*CI*	*P* Value
Age						
70–76 years					1	
>76 years	0.716	0.125	32.975	2.047	(1.603,2.614)	**<0.001**
Histological Grade						
Grade I					1	
Grade II–III	0.723	0.179	16.384	2.060	(1.452,2.923)	**<0.001**
Chemotherapy Status						
No Chemotherapy					1	
Chemotherapy	−0.027	0.128	0.043	0.974	(0.757,1.252)	0.835
Number of Positive Axillary Lymph Nodes						
1 Node					1	
2 Nodes	0.289	0.108	7.096	1.335	(1.079,1.651)	**0.008**
Tumor Size						
≤1.4 cm					1	
1.4 cm-5 cm	0.511	0.113	20.598	1.667	(1.337,2.079)	**<0.001**
Molecular Subtype						
HR + ,HER2-					1	
HR + ,HER2+	−0.148	0.175	0.716	0.862	(0.612,1.215)	0.398
HR-,HER2+	−0.001	0.331	0.000	0.999	(0.523,1.910)	0.998
HR-,HER2-	0.768	0.204	16.440	2.289	(1.534,3.416)	**<0.001**
Annual Income (×10^3^, USD)						
<55					1	
55 ~ 74	−0.042	0.117	0.130	0.959	(0.763,1.205)	0.718
≥75	−0.435	0.139	9.844	0.647	(0.493,0.849)	**0.002**
Axillary Treatment Modality						
SLNB+RT					1	
SLNB without RT	0.628	0.162	14.969	1.873	(1.363,2.575)	**<0.001**
ALND+ RT	0.139	0.153	0.768	0.363	(0.852,1.549)	0.363
ALND without RT	0.572	0.161	12.552	1.771	(1.291,2.430)	**<0.001**

### Risk-stratified subgroup survival analysis

In the high-risk group, 5-year BCSS rates were: SLNB+RT 91.3%, SLNB without RT 76.6%, ALND+RT 91.5%, and ALND without RT 84.5%. In the intermediate-risk group, rates were: SLNB+RT 96.9%, SLNB without RT 92.3%, ALND+RT 94.9%, and ALND without RT 92.7%. For both high- and intermediate-risk groups, Kaplan–Meier analysis showed significant survival benefit with SLNB+RT (Fig 3, Fig 4; Table 5).In the low-risk group, 5-year BCSS rates were: SLNB+RT 96.3%, SLNB without RT 95.2%, ALND+RT 96.5%, and ALND without RT 94.3%, with no statistically significant differences among the four groups (*P* > 0.05; Fig 5, Table 5).

**Table 5:** Pairwise comparisons of BCSS among axillary treatment modalities by risk group.

Low-Risk Group						
Axillary Treatment Modality	SLNB+RT (96.3%)		SLNB without RT (95.2%)		ALND+ RT (96.5%)	
	*χ* ^*2*^	*P* value	*χ* ^*2*^	*P* value	*χ* ^*2*^	*P* value
SLNB without RT(95.2%)	0.816	0.366	–	–	–	–
ALND+ RT(96.5%)	0.000	0.985	0.590	0.442	–	–
ALND without RT(94.3%)	1.522	0.217	0.170	0.680	1.607	0.205
Intermediate-Risk Group						
Axillary Treatment Modality	SLNB+ RT (96.9%)		SLNB without RT (92.3%)		ALND+ RT (94.9%)	
	*χ* ^*2*^	*P* value	*χ* ^*2*^	*P* value	*χ* ^*2*^	*P* value
SLNB without RT(92.3%)	8.158	**0.004**	–	–	–	–
ALND+ RT(94.9%)	1.032	0.312	4.260	**0.039**	–	–
ALND without RT(92.7%)	5.909	**0.015**	0.170	0.680	1.994	0.158
High-Risk Group						
Axillary Treatment Modality	SLNB+ RT (91.3%)		SLNB without RT (76.6%)		ALND+ RT (91.5%)	
	*χ* ^*2*^	*P* value	*χ* ^*2*^	*P* value	*χ* ^*2*^	*P* value
SLNB without RT(76.6%)	12.676	**<0.001**	–	–	–	–
ALND+ RT (91.5%)	0.002	0.965	12.394	**<0.001**	–	–
ALND without RT (84.5%)	11.618	**0.015**	0.046	0.830	12.227	**<0.001**

**Fig 3:**
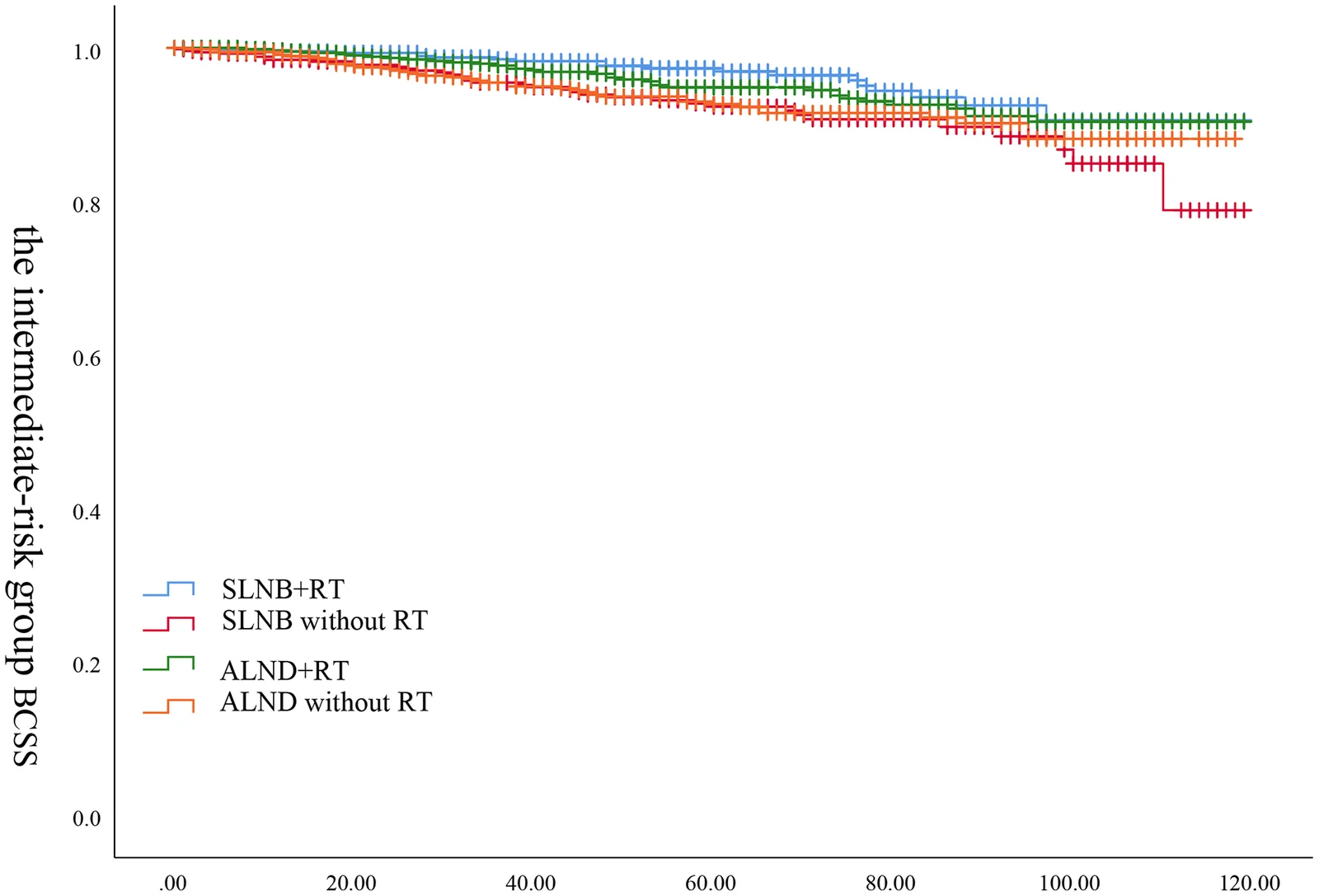
Kaplan–Meier curves for BCSS in the intermediate-risk group.

**Fig 4:**
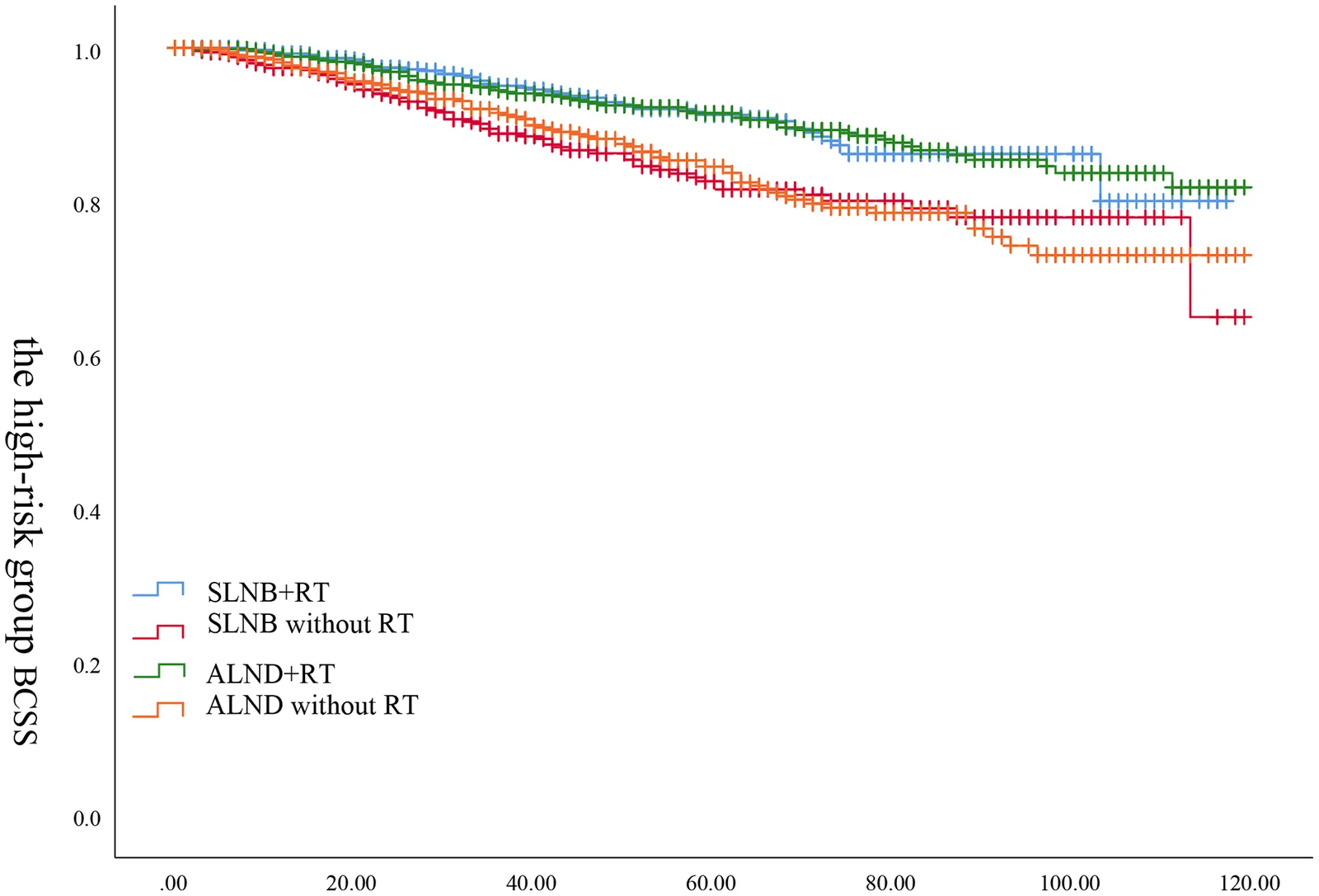
Kaplan–Meier curves for BCSS in the high-risk group.

**Fig 5:**
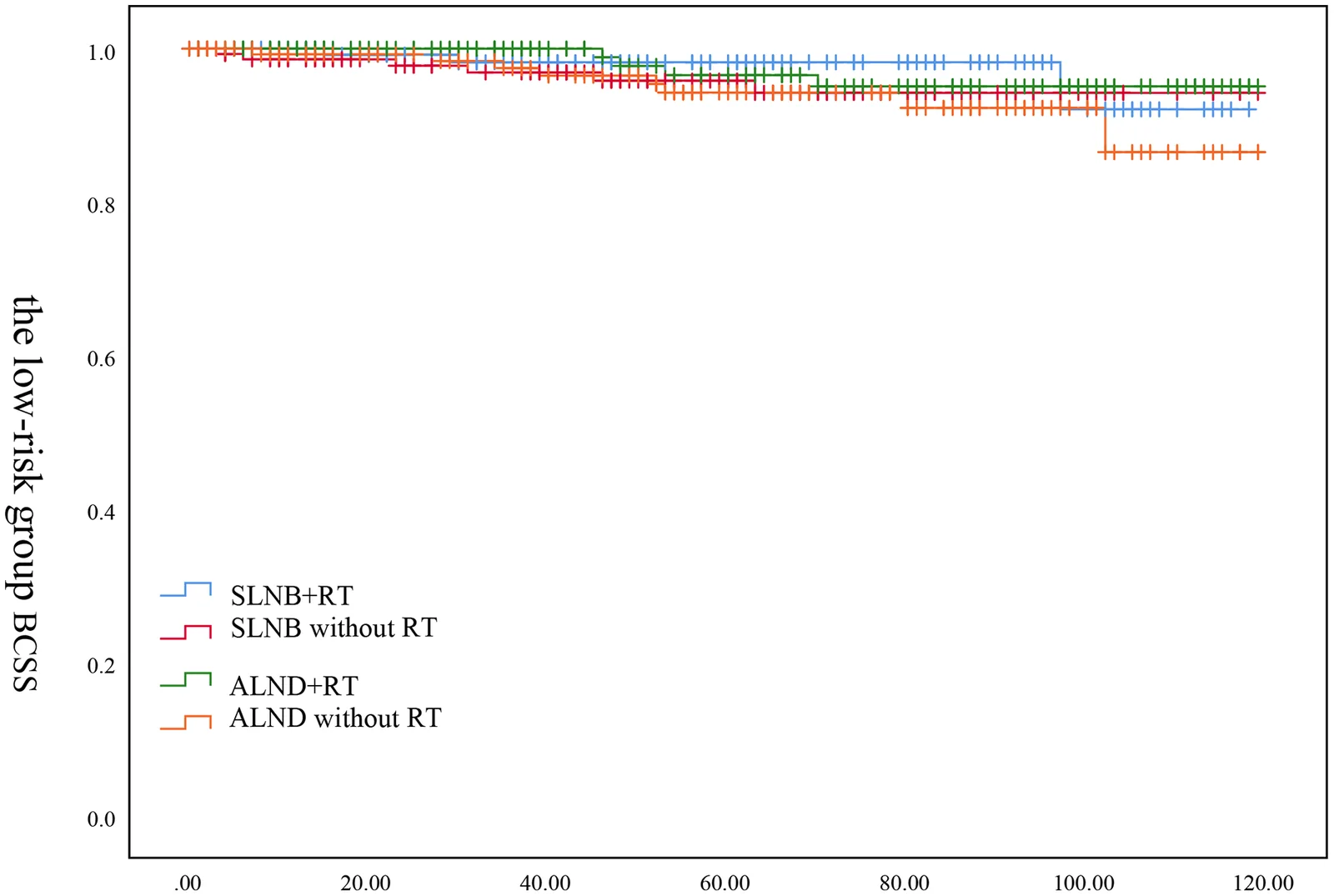
Kaplan–Meier curves for BCSS in the low-risk group.

### Clinicopathological characteristics of the four treatment groups (Independent Chinese descriptive case‑series)

The independent Chinese descriptive case‑series comprised 106 patients, with no significant differences in baseline characteristics among the four axillary treatment groups (*P* > 0.05 for all; Table 6).

**Table 6:** Clinicopathological characteristics across four axillary treatment groups.

Clinicopathological Characteristics	SLNB + RT (n = 32)	ALND + RT (n = 29)	ALND without RT (n = 19)	SLNB without RT (n = 26)	*χ²*	*P* value
Age					0.12	0.988
70–76 years	22 (68.8%)	19 (65.5%)	13 (68.4%)	18 (69.2%)		
>76 years	10 (31.2%)	10 (34.5%)	6 (31.6%)	8 (30.8%)		
Tumor Size					0.78	0.854
**<**1.4 cm	13 (40.6%)	10 (34.5%)	7 (36.8%)	8 (30.8%)		
1.4 ~ 5 cm	19 (59.4%)	19 (65.5%)	12 (63.2%)	18 (69.2%)		
Histological Grade					1.89	0.596
Grade I	8 (25.0%)	7 (24.1%)	3 (15.8%)	4 (15.4%)		
Grade II–III	24 (75.0%)	22 (75.9%)	16 (84.2%)	22 (84.6%)		
Number of Positive Axillary Lymph Nodes					0.98	0.805
1 Node	20 (62.5%)	18 (62.1%)	10 (52.6%)	14 (53.8%)		
2 Nodes	12 (37.5%)	11 (37.9%)	9 (47.4%)	12 (46.2%)		
Molecular Subtype					5.12	0.824
Luminal A subtype	14 (43.8%)	12 (41.4%)	7 (36.8%)	9 (34.6%)		
Luminal B subtype	12 (37.5%)	11 (37.9%)	7 (36.8%)	8 (30.8%)		
HER2-positive subtype	4 (12.5%)	4 (13.8%)	3 (15.8%)	5 (19.2%)		
Triple-negative subtype	2 (6.2%)	2 (6.9%)	2 (10.5%)	4 (15.4%)		
T Stage					0.67	0.881
T_1_	19 (59.4%)	16 (55.2%)	10 (52.6%)	13 (50.0%)		
T_2_	13 (40.6%)	13 (44.8%)	9 (47.4%)	13 (50.0%)		
Breast Surgery Type					1.56	0.669
Breast-Conserving Surgery	16 (50.0%)	13 (44.8%)	8 (42.1%)	9 (34.6%)		
Unilateral Mastectomy	16 (50.0%)	16 (55.2%)	11 (57.9%)	17 (65.4%)		

### Multivariate logistic regression for 5-year LRR

Multivariate logistic regression identified the following independent risk factors for 5-year LRR: age > 76 years (OR = 2.116, 95% CI: 1.324–3.382, *P* < 0.001), grade II–III (OR = 2.083, 95% CI: 1.271–3.416, *P* < 0.001), two positive nodes (OR = 1.352, 95% CI: 1.041–1.756, *P* = 0.023), tumor size 1.4–5 cm (OR = 1.715, 95% CI: 1.302–2.258, *P* < 0.001), triple-negative subtype (OR = 2.341, 95% CI: 1.486–3.687, *P* < 0.001), SLNB without RT (OR = 1.928, 95% CI: 1.415–2.626, *P* < 0.001), and ALND without RT (OR = 1.814, 95% CI: 1.307–2.515, *P* < 0.001; Table 7).

**Table 7:** Multivariate logistic regression for 5-year LRR.

Clinicopathological Characteristics	Regression Coefficient	Standard Error (SE)	Wald *χ²*	*OR (95%CI)*	*P* Value
Age					
70–76 years				1	
>76 years	0.749	0.241	9.67	2.116 (1.324 ~ 3.382)	<0.001
Histological Grade					
Grade I				1	
Grade II–III	0.734	0.252	8.47	2.083 (1.271 ~ 3.416)	<0.001
Number of Positive Axillary Lymph Nodes					
1 Node				1	
2 Nodes	0.302	0.134	5.07	1.352 (1.041 ~ 1.756)	0.023
Tumor Size					
≤1.4 cm				1	
1.4 ~ 5 cm	0.539	0.142	14.42	1.715 (1.302 ~ 2.258)	<0.001
Molecular Subtype					
Other Subtypes				1	
Triple-negative Subtype	0.850	0.231	13.54	2.341 (1.486 ~ 3.687)	<0.001
Axillary Treatment Modality					
SLNB + RT				1	–
ALND + RT	0.010	0.072	0.02	1.010 (0.878 ~ 1.161)	0.892
ALND without RT	0.596	0.168	12.63	1.814 (1.307 ~ 2.515)	<0.001
SLNB without RT	0.656	0.159	17.04	1.928 (1.415 ~ 2.626)	<0.001

### Local regional recurrence-free analysis by Risk Group

In the total population, the 5-year local regional recurrence-free survival curves of different axillary treatment methods are shown in Fig 6. The recurrence-free survival rates of SLNB+RT and SLNB without RT are comparable, while those of ALND without RT are the lowest (χ² = 12.776，P = 0.005). In the low-risk group, no recurrences occurred within 5 years across all four treatment arms. In the intermediate-risk group, 5-year LRR rates were: SLNB+RT 6.67%, SLNB without RT 33.33%, ALND+RT 7.69%, and ALND without RT 20.00%. In the high-risk group, rates were: SLNB+RT 7.69%, SLNB without RT 50.00%, ALND+RT 7.70%, and ALND without RT 42.86% (Table 8).

**Table 8:** Five-year LRR rates by risk group and axillary treatment modality.

Low-Risk Group						
Axillary Treatment Modality	SLNB+RT (0)		SLNB without RT (0)		ALND+ RT (0)	
	*χ* ^*2*^	*P* value	*χ* ^*2*^	*P* value	*χ* ^*2*^	*P* value
SLNB without RT (0)	0.000	1.000	–	–	–	–
ALND+ RT(0)	0.000	1.000	0.000	1.000	–	–
ALND without RT(0)	0.000	0.000	0.000	1.000	0.000	1.000
Intermediate-Risk Group						
Axillary Treatment Modality	SLNB+ RT (6.67%)		SLNB without RT (33.33%)		ALND+ RT (7.69%)	
	*χ* ^*2*^	*P* value	*χ* ^*2*^	*P* value	*χ* ^*2*^	*P* value
SLNB without RT(33.33%)	5.891	**0.015**	–	–	–	–
ALND+ RT (7.69%)	0.016	0.899	4.113	**0.043**	–	–
ALND without RT (20.00%)	4.220	**0.040**	0.169	0.681	3.987	0.046
High-Risk Group						
Axillary Treatment Modality	SLNB+ RT (7.69%)		SLNB without RT (50.00%)		ALND+ RT (7.70%)	
	*χ* ^*2*^	*P* value	*χ* ^*2*^	*P* value	*χ* ^*2*^	*P* value
SLNB without RT (50.00%)	12.441	**<0.001**	–	–	–	–
ALND+ RT (7.70%)	0.003	0.956	12.306	**<0.001**	–	–
ALND without RT (42.86%)	11.582	**<0.001**	0.044	0.834	11.437	**<0.001**

**Fig 6:**
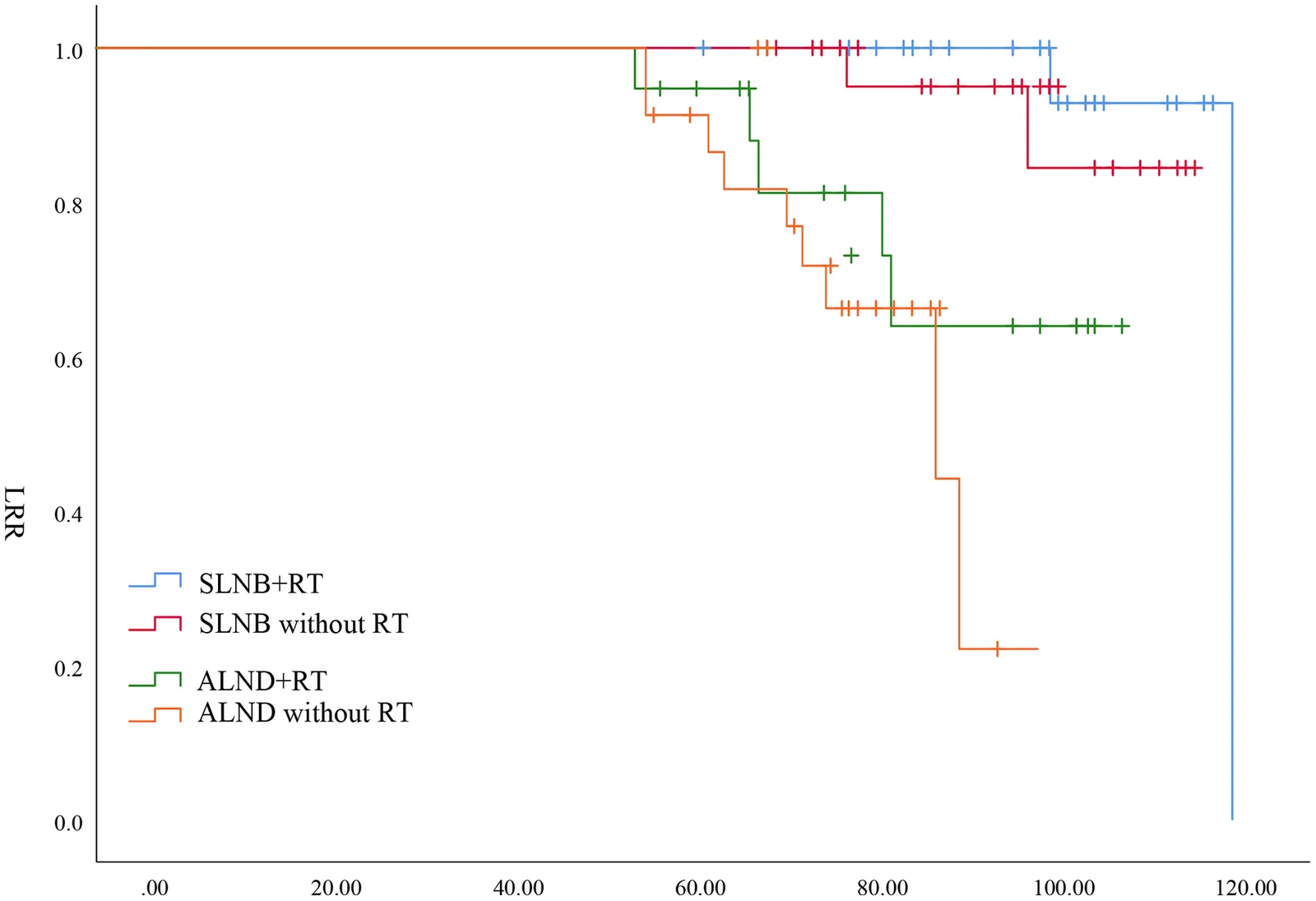
5-year local regional recurrence-free survival curves of different axillary treatment methods.

## Discussion

Our findings demonstrate that the benefit of different axillary management strategies is highly heterogeneous among elderly patients (≥70 years) with early-stage breast cancer and 1–2 positive lymph nodes. High- and intermediate-risk patients derive significant survival benefit from SLNB combined with RT, whereas low-risk patients show comparable outcomes across all four treatment modalities. These results provide important real-world evidence to guide individualized axillary management in this understudied population.

### Overall Axillary Management Strategy

In the overall cohort analysis, SLNB+RT achieved a 5-year BCSS of 94.4%, significantly superior to the other three modalities, while ALND+RT yielded 93.3%, with no statistically significant difference between these two regimens. This finding aligns with the 2024 international multicenter phase III randomized controlled trial published in the *New England Journal of Medicine*, which enrolled 2,766 patients with sentinel node metastases and demonstrated non-inferiority of omitting ALND (5-year recurrence-free survival: 89.7% vs. 88.7%; HR = 0.89) [12]. Similarly, Li et al., using SEER data, found no significant association between ALND and BCSS in T1–2 breast cancer patients with 1–2 positive nodes [13]. The 2025 ASCO guideline update further expanded SLNB indications, supporting ALND omission in more patients [14].

Critically, regardless of surgical approach, patients who did not receive RT had significantly worse survival outcomes, underscoring the vital role of RT in this population [15]. The Z0011 trial established the standard for omitting ALND in patients with 1–2 positive sentinel nodes who undergo breast-conserving surgery. Our findings extend this paradigm to elderly patients, demonstrating that SLNB+RT achieves outcomes at least equivalent to ALND+RT, even with established nodal metastases. This may reflect the relatively indolent tumor biology often seen in older patients, the efficacy of RT in controlling axillary micrometastases [16], and the morbidity associated with ALND offsetting its potential survival benefit.

### Clinical Significance of Risk Stratification

A key innovation of our study is the risk-stratified subgroup analysis based on five independent adverse prognostic factors identified by multivariate Cox regression. This model complements prior work, such as the study by Wu et al. (2023), which identified two positive sentinel nodes (OR = 3.601), T2 stage (OR = 4.681), and lymphovascular invasion (OR = 3.781) as independent predictors of non-sentinel node metastasis [17].

For low-risk patients (no adverse factors), 5-year BCSS exceeded 94% across all four treatment arms with no significant intergroup differences. This suggests that for elderly patients with favorable tumor biology, even in the presence of 1–2 positive nodes, aggressive locoregional treatment does not confer additional survival benefit. SLNB alone suffices, and further ALND or RT may represent overtreatment. Supporting this, an Israeli study reported that in patients aged ≥70 years (median 81 years) with early-stage luminal breast cancer, omitting both SLNB and RT was feasible, with a 3.9-year recurrence rate of only 7% [18]. For low-risk elderly patients, minimizing treatment-related morbidity and prioritizing quality of life should be paramount.

For intermediate-risk patients (one adverse factor), SLNB+RT achieved a 5-year BCSS of 96.9%, significantly superior to SLNB without RT (92.3%) and ALND without RT (92.7%), and comparable to ALND+RT (94.9%). These results indicate that RT is the key determinant of survival benefit in this group, whereas ALND provides no additional advantage. A Japanese study similarly found that in patients ≥75 years with HR-negative breast cancer, omitting axillary surgery, RT, or chemotherapy did not significantly affect prognosis [19]. Thus, SLNB with postoperative RT represents the optimal strategy for intermediate-risk patients, avoiding ALND-related complications while ensuring adequate local control.

For high-risk patients (2–5 adverse factors), survival differences were most pronounced. SLNB+RT and ALND+RT yielded nearly identical 5-year BCSS rates (91.3% vs. 91.5%), both significantly superior to the non-irradiated groups. Notably, SLNB without RT had the worst 5-year BCSS (76.6%) among all subgroups, highlighting a critical clinical caveat: RT should never be omitted solely based on age in high-risk patients. A 2022 study from the PLA General Hospital also confirmed that in node-negative patients, SLNB was associated with superior outcomes compared to ALND [20]. Although SLNB avoids ALND complications, RT is essential to optimize survival. Reassuringly, even in high-risk patients, SLNB+RT achieved equivalent outcomes to ALND+RT, suggesting that ALND can be safely avoided across all risk strata when RT is administered.

### Re-evaluating the role of radiotherapy in elderly breast cancer

One of the most striking findings of our study is the profound survival impact of RT. Regardless of surgical approach, patients who did not receive RT had significantly worse BCSS. In multivariate analysis, SLNB without RT (HR = 1.873) and ALND without RT (HR = 1.771) conferred greater risk than even two positive nodes (HR = 1.335). This suggests that, in elderly patients with 1–2 positive nodes, the importance of RT may surpass that of the extent of axillary surgery.

These results are contextualized by prior studies with seemingly contradictory findings. Luo et al., analyzing 7,052 patients aged ≥70 years with T1–2N1 breast cancer from SEER, found that postmastectomy RT (PMRT) provided survival benefit only in highly selected subgroups—specifically, those with three positive nodes, tumors 2–5 cm, and no chemotherapy [21]. Another SEER study of 106,553 elderly patients reported that RT in T1–2N1 patients undergoing mastectomy was associated with worse survival [22]. The apparent discrepancy likely reflects patient selection: our study includes both mastectomy and breast-conserving surgery patients, and the benefit of RT may vary by surgical context. Indeed, Lu et al. (2025) reported that in 356 SLN-positive patients who did not undergo ALND, regional nodal irradiation (RNI) eliminated recurrences, while the no-RNI group had a 3.5% recurrence rate, with tumors >2 cm and triple-negative status deriving the most benefit [23]. The Italian LISEN trial (2022) also confirmed that in patients meeting Z0011 criteria, whole-breast RT without axillary irradiation achieved 5-year DFS of 93.6% and OS of 96.9%, with extremely low regional recurrence [24].

Historically, the value of RT in elderly patients has been debated. The CALGB 9343 trial demonstrated that in patients aged ≥70 years with ER-positive, node-negative stage I breast cancer, RT reduced local recurrence but did not impact OS [25]. However, that study involved node-negative patients; our findings indicate that for node-positive patients, RT significantly improves both local control and BCSS, a benefit that may be particularly important given the relatively immunocompromised state of many elderly individuals.

Martelli et al., in a 15-year randomized controlled trial, showed that omitting ALND did not affect survival in patients aged 65–80 years with T1N0 breast cancer [26], extending the principle of axillary de-escalation. McMahon et al. demonstrated that SLNB is safe and feasible in elderly patients and significantly influences adjuvant treatment decisions [27]. A 2023 study from Cedars-Sinai Medical Center reported that among 881 low-risk elderly patients, RT rates declined from 58% in 2012 to 36% in 2022, and SLNB rates from 86% to 56%, without compromising survival [28]. Similarly, data from six Italian cancer centers (2022) showed that although de-escalation rates for axillary staging and RT remain low (3.3% and 8.6%, respectively), patients aged 75–79 years and those undergoing mastectomy were more likely to receive de-escalated treatment [29].

Domestic studies have also contributed to this evidence base. Liu et al. found that SLNB may be safely omitted in patients aged ≥70 years with T1–2, HR-positive, clinically node-negative disease [30]. Zhou et al. emphasized that elderly breast cancer patients have lower malignancy grades but more comorbidities, necessitating individualized treatment [31]. Our study advances this field by providing risk-stratified, evidence-based recommendations for optimal axillary management in this population.

### Study limitations

Several limitations should be acknowledged. First, as a retrospective study, despite PSM to reduce selection bias, residual confounding from unmeasured variables cannot be excluded. Second, the SEER database lacks data on treatment-related complications such as lymphedema and quality of life, precluding safety comparisons across modalities. Third, axillary surgical grouping (SLNB versus ALND) was defined by total number of examined lymph nodes as a proxy measure, since SEER lacks direct procedural codes distinguishing SLNB and ALND. Misclassification bias may exist: rarely, technically extensive SLNB may harvest ≥6 nodes, while incomplete ALND may retrieve fewer than six nodes. Such misclassification could dilute true effect sizes towards null. Nevertheless, this cut‑off has been repeatedly adopted in peer‑reviewed SEER breast‑cancer studies, based on AJCC axillary dissection criteria and published large‑cohort evidence.. Fourth, SEER database lacks granular systemic‑therapy information including endocrine therapy adherence/duration and HER2‑targeted therapy usage. These variables strongly influence breast‑cancer‑specific survival and constitute important unmeasured confounding factors. We could not incorporate these covariates into multivariate Cox models. Therefore, associations observed between axillary treatment strategies and BCSS should be interpreted cautiously. The conclusions are hypothesis‑generating and require further prospective validation. Fifth, our SEER study population consists of patients diagnosed between 2010‑2019. Although data extraction occurred in February 2023, we selected this diagnosis window to guarantee complete 5‑year follow‑up for BCSS and locoregional recurrence endpoints. More recently‑diagnosed cases (2020 onwards) in SEER do not yet have mature 5‑year survival data. Therefore our findings reflect real‑world practice from 2010‑2019, and contemporary axillary de‑escalation patterns after 2020 may not be fully represented. Caution should be applied when extrapolating these results to very recent clinical practice. Sixth, our tumour‑size binary cut‑off point of 1.4 cm originates from a prior study limited to breast‑conserving‑surgery patients. We applied this threshold to both mastectomy and breast‑conserving‑surgery participants without separate TNM T‑subgroup stratification (T1a, T1b, T2). Future work should perform separate stratified analyses according to formal AJCC T subcategories for mastectomy versus breast‑conserving‑surgery populations. Finally, although we included a independent Chinese descriptive case‑series, its small sample size (n = 106) limits generalizability, and the primary analysis was predominantly based on U.S. patients. External validation in larger, diverse populations is warranted.

### Clinical implications and future directions

Our findings have direct clinical implications. For elderly patients (≥70 years) with early-stage breast cancer and 1–2 positive nodes, we recommend an individualized, risk-stratified approach:

- Low-risk patients (0 risk factors): SLNB alone is sufficient; ALND and RT may be omitted to minimize morbidity.
- Intermediate-risk patients (1 risk factor): SLNB combined with postoperative RT is recommended; ALND is unnecessary.
- High-risk patients (2–5 risk factors): SLNB combined with RT is strongly recommended; ALND does not confer additional survival benefit.

Prospective randomized trials are needed to validate these recommendations. Future research should incorporate geriatric assessment tools, patient-reported outcomes, and quality-of-life measures to ensure that survival gains are balanced against functional preservation and overall well-being. As the field of geriatric oncology advances, the goal should be individualized care that achieves both optimal oncologic outcomes and maintained quality of life.
