# Supplementary material for: Effect of different axillary management strategies on survival in elderly patients (≥70 Years) with early-stage breast cancer and 1–2 positive lymph nodes, stratified by risk factors
Source: PLoS One. 2026 Nov 11;21(11):e0353331. doi: 10.1371/journal.pone.0353331 (PMC13754101; doi:10.1371/journal.pone.0353331)
Supplement: Supplementary file 1 [file pone.0353331.s001.docx]

# **Reviewer Comments**

**Manuscript Title:** PONE-D-26-30832 — "Effect of Different Axillary Management Strategies on Survival in Elderly Patients (≥70 Years) with Early-Stage Breast Cancer and 1–2 Positive Lymph Nodes, Stratified by Risk Factors"

## **Overall Recommendation: Major Revision**

This manuscript addresses a clinically important and understudied question, i.e., individualised axillary management in elderly (≥70 years) patients with early-stage breast cancer and 1–2 positive nodes using a well-powered, propensity-matched SEER cohort with a real-world validation component. The study design, risk-stratification approach, and overall conclusions are sound and clinically meaningful. However, certain methodological issues require clarification and revision, most notably: the absence of systemic therapy data as a potential confounder, the dated nature of the underlying cohort relative to current clinical practice, the proxy-based (rather than procedure-verified) definition of SLNB versus ALND, and ambiguity regarding how the validation cohort was incorporated into the propensity score matching. I recommend the authors address these points in a revised submission.

## **Comment 1: Missing systemic therapy data (major confounder)**

The manuscript does not report receipt of HER2-targeted therapy or endocrine therapy adherence/duration, both of which are major, well-established determinants of breast cancer-specific survival (BCSS), particularly relevant as a substantial proportion of the cohort is HR+/HER2+ or HR−/HER2+. Without accounting for systemic therapy exposure, it is difficult to isolate the independent contribution of axillary management strategy (SLNB vs. ALND, ±RT) to the observed survival differences. The authors should clarify whether these data were available in the SEER extract and, if so, incorporate them into the multivariate models; if unavailable, this should be explicitly acknowledged as a significant limitation, as it may confound the central conclusions regarding RT and surgical de-escalation.

## **Comment 2: Outdated cohort**

The SEER data were extracted on 02/03/2023, yet the manuscript is being submitted for review roughly three years later. This gap is clinically significant because axillary de-escalation practices (e.g., omission of ALND per updated Z0011-consistent recommendations, and evolving RT indications) have changed meaningfully in just the last two to three years. Findings based on a 2010–2019 diagnosis cohort with a 2023 data pull may not adequately reflect current practice patterns or contemporary outcomes, and the discussion should more explicitly address how this lag may affect the generalizability and clinical applicability of the recommendations being made. The authors should comment on why publication was delayed relative to data extraction, and whether more recent SEER data could be incorporated or at least discussed.

## **Comment 3: SLNB/ALND classification is a proxy, not a true procedural classification**

Because the SEER database does not record the specific axillary surgical procedure performed, the authors have operationally defined SLNB as removal of 1–5 lymph nodes and ALND as removal of ≥6 nodes. This is a proxy definition and not a verified classification of actual surgical intent or technique. Nodal yield can vary independent of procedure type (e.g., an anatomically extensive SLNB, or a technically limited ALND), which introduces a risk of misclassification bias that could affect the comparison at the heart of this study. The authors should more directly acknowledge this as a methodological limitation (beyond the citation-based justification currently provided) and discuss its potential direction and magnitude of impact on the reported associations between axillary strategy and survival.

## **Comment 4: Clarification needed on PSM cohort composition (SEER vs. validation cohort)**

The Methods state that PSM was applied to "the combined cohort" of SEER (n=8,977) and validation (n=106) patients, yielding 5,858 matched patients. However, the pre-PSM group totals reported in Table 1 (5,447 + 3,530 = 8,977) correspond exactly to the SEER cohort alone, and Table 6 presents baseline characteristics of the 106 validation patients as a separate, unmatched four-group comparison with no accompanying survival analysis. This raises ambiguity as to whether the validation cohort was in fact included in the PSM procedure or analysed as a wholly independent dataset. The authors should clarify: (a) whether the 106 Chinese patients were included in the propensity score matching process, and (b) if they were analysed separately, the rationale for doing so, and how this affects the claim that PSM was applied to a "combined" cohort. If analysed separately, the term "validation cohort" is misleading, since no independent validation of the survival findings is actually presented for this subset.
