# Supplementary material for: Effect of different axillary management strategies on survival in elderly patients (≥70 Years) with early-stage breast cancer and 1–2 positive lymph nodes, stratified by risk factors
Source: PLoS One. 2026 Nov 11;21(11):e0353331. doi: 10.1371/journal.pone.0353331 (PMC13754101; doi:10.1371/journal.pone.0353331)
Supplement: Supplementary file 2 [file pone.0353331.s003.docx]

**Response to Reviewers**

Manuscript ID: **PONE‑D‑26‑30832**

Manuscript Title: *Effect of Different Axillary Management Strategies on Survival in Elderly Patients (≥70 Years) with Early‑Stage Breast Cancer and 1–2 Positive Lymph Nodes, Stratified by Risk Factors*

Dear Academic Editor Dr. Sanjay Kumar Yadav and respected Reviewers,

We sincerely thank you and the reviewers for your thorough evaluation, constructive and insightful comments on our manuscript. We have carefully addressed **every comment point‑by‑point**, revised the manuscript accordingly, and prepared two versions of manuscript files: *Revised Manuscript with Track Changes* and clean *Manuscript*. Our raw analysis dataset has been deposited in Mendeley Data (DOI: **10.17632/55brtt8hw9.1**) to fulfill PLOS ONE data availability requirements.

Below please find our detailed point‑by‑point responses. Page and line numbers refer to the **track‑changed revised manuscript**.

**Editorial Office Mandatory Requirements**

**Comment 1. Funding information handling**

Thank you for stating the following in the Acknowledgments Section of your manuscript: “Supported by Health Commission of Sichuan Province Medical Science and Technology Program (25LCYJ64)”. Funding information should NOT appear in Acknowledgments section; only fill in online submission form Funding Statement. Please state the role of funders.

**Response**: We fully understand this PLOS ONE requirement.

1. We have **removed all funding‑related text from the manuscript main body and acknowledgments section** (Revised Manuscript, Page 1).
2. Role of funders statement: *“The funders had no role in study design, data collection and analysis, decision to publish, or preparation of the manuscript.”*
3. We will fill the funding grant number and above funder‑role statement in the online submission form as instructed.

**Comment 2. Data Availability Statement, minimal dataset requirement**

Please confirm your submission contains minimal dataset for replication; provide DOI/link for datasets. If restrictions exist, explain.

**Response**: Our complete minimal dataset supporting all statistical analyses has been publicly deposited to **Mendeley Data**:

DOI: 10.17632/55brtt8hw9.1 URL: <https://data.mendeley.com/datasets/55brtt8hw9/1>

We have updated the **Data Availability Statement** in revised manuscript (Page 2, Methods section):

“All minimal datasets required to replicate findings of this study are publicly available at Mendeley Data, DOI:10.17632/55brtt8hw9.1. The Chinese single‑center de‑identified patient dataset is included within the Mendeley Data repository.”

All summary statistics, table values, KM curve source data points are provided in the uploaded repository. No ethical restrictions apply to these de‑identified data.

**Comment3. Figures 1‑6 resolution is insufficient, upload new high‑quality figure files**

Please upload new copy of Figures1‑6 with sufficient resolution following PLOS ONE figure guidelines.

**Response**: We have regenerated all six Kaplan‑Meier survival figures following PLOS ONE illustration specifications (300 DPI, vector‑friendly format, readable axis labels, legend, tick marks). New figure files will be uploaded during resubmission.

**Comment4. Suggest depositing laboratory protocol to protocols.io (optional recommendation)**

**Response**: Thank you for this suggestion. Our analysis workflow is fully described within manuscript Methods section. For this retrospective registry study, we will not deposit a separate protocol.io entry at this stage, but all R/SPSS analysis logic is described in Methods, and raw dataset is available in Mendeley Data for full reproducibility.

**Comment5. ORCID for co‑authors**

Encourage all co‑authors to register and verify ORCID iDs.

**Response**: We will remind all co‑authors to complete ORCID registration and verification in Editorial Manager before final acceptance.

**Reviewer #1 Comments**

Overall comment: This manuscript addresses a clinically important and understudied question, i.e., individualised axillary management in elderly (≥70 years) patients with early‑stage breast cancer and 1–2 positive nodes using a well‑powered, propensity‑matched SEER cohort with a real‑world validation component. The study design, risk‑stratification approach, and overall conclusions are sound and clinically meaningful. However, certain methodological issues require clarification and revision.

**Comment 1‑1: Missing systemic therapy data as major confounder**

The manuscript does not report receipt of HER2‑targeted therapy or endocrine therapy adherence/duration, both of which are major, well‑established determinants of breast cancer‑specific survival (BCSS). Without accounting for systemic therapy exposure, it is difficult to isolate independent contribution of axillary management strategy. Clarify whether these data were available; if unavailable, explicitly acknowledge as significant limitation.

**Response**: We greatly appreciate this critical comment. The SEER registry database **does NOT capture granular information regarding endocrine therapy adherence, duration, or HER2‑targeted agent administration** for our study period, only simple yes/no chemotherapy flag. This represents an important unmeasured confounder.

We have added explicit discussion of this limitation in **Discussion‑Limitations subsection**. Added text excerpt:

“Fourth, SEER database lacks granular systemic‑therapy information including endocrine therapy adherence/duration and HER2‑targeted therapy usage. These variables strongly influence breast‑cancer‑specific survival and constitute important unmeasured confounding factors. We could not incorporate these covariates into multivariate Cox models. Therefore, associations observed between axillary treatment strategies and BCSS should be interpreted cautiously. The conclusions are hypothesis‑generating and require further prospective validation.”

**Comment 1‑2: Outdated cohort; data extraction lag relative to current clinical practice**

SEER data extracted Feb‑03‑2023; manuscript reviewed ~3 years later. Axillary de‑escalation practice patterns have evolved. Address generalizability; comment on publication delay; discuss whether newer SEER data could be incorporated.

**Response**: Thank you for pointing this out. Diagnosis years of our SEER cohort are **2010‑2019**. Although we performed data pull in Feb 2023, diagnosis time window remains fixed for our study aim. We could not re‑extract newer SEER diagnosis years (2020‑2022): SEER registry requires ~24 months lag for complete survival follow‑up; patients diagnosed after 2019 lack sufficient 5‑year follow‑up for calculating BCSS and locoregional recurrence endpoints required for our risk‑stratified analysis. Adding newer cases would render our primary 5‑year endpoints unreliable.

We added expanded text in Limitations section :

“Fifth, our SEER study population consists of patients diagnosed between 2010‑2019. Although data extraction occurred in February 2023, we selected this diagnosis window to guarantee complete 5‑year follow‑up for BCSS and locoregional recurrence endpoints. More recently‑diagnosed cases (2020 onwards) in SEER do not yet have mature 5‑year survival data. Therefore our findings reflect real‑world practice from 2010‑2019, and contemporary axillary de‑escalation patterns after 2020 may not be fully represented. Caution should be applied when extrapolating these results to very recent clinical practice.”

**Comment 1‑3: SLNB / ALND classification is proxy definition (by number of resected nodes), not procedure‑verified variable**

SEER does not record actual axillary surgical intent; we use nodal yield as surrogate. Nodal yield varies independent of operation type, creates misclassification bias. Acknowledge this limitation more directly and discuss potential bias magnitude / direction.

**Response:** This is a well‑recognised limitation for SEER‑based breast‑cancer axillary‑surgery studies. Our proxy definition (SLNB = 1‑5 lymph nodes removed; ALND ≥ 6 nodes removed) follows widely‑published prior SEER‑based breast‑cancer publications, which we cite in manuscript.

We have strengthened this limitation statement in Limitations (Page 28):

“Third, axillary surgical grouping (SLNB versus ALND) was defined by total number of examined lymph nodes as a proxy measure, since SEER lacks direct procedural codes distinguishing SLNB and ALND. Misclassification bias may exist: rarely, technically extensive SLNB may harvest ≥6 nodes, while incomplete ALND may retrieve fewer than six nodes. Such misclassification could dilute true effect sizes towards null. Nevertheless, this cut‑off has been repeatedly adopted in peer‑reviewed SEER breast‑cancer studies, based on AJCC axillary dissection criteria and published large‑cohort evidence.”

**Comment 1‑4: Clarify PSM cohort composition: SEER vs Chinese validation cohort**

Methods state “PSM applied to combined cohort”, but table 1 totals correspond only to SEER n=8977. The n=106 Chinese patients are separate unmatched descriptive cohort with no survival analysis. Is the 106‑patient cohort included in PSM? If analysed separately, “validation cohort” term may be misleading. Clarify rationale.

**Response**: Thank you for catching this important wording ambiguity. **The 106 Chinese single‑centre patients were NOT included in propensity‑score matching.** PSM analysis was performed exclusively on the SEER‑database cohort (n=8977). The Chinese 106‑patient dataset serves only as an **external descriptive case series**, for baseline feature comparison; we did NOT perform survival / recurrence statistical validation on this small sample due to limited event numbers. The original manuscript phrase “combined cohort” was inaccurate wording.

We have revised:

Methods‑Study Population section (Page 3): removed the misleading wording “combined cohort”. Clearly write: *“Propensity score matching was performed exclusively within the SEER‑database cohort (n = 8 977). An independent Chinese single‑centre case series (n = 106) was used for descriptive baseline comparison only and was not incorporated into PSM”*

The term “validation cohort” has been replaced throughout manuscript with “independent Chinese descriptive case‑series” to eliminate misinterpretation.

**Reviewer #2 Comments**

Overall: The study has been conducted in a very meticulous manner. However there are some doubts that are of concern.

**Comment 2‑1: Tumor size cut‑off 1.4–5 cm adopted from reference‑8 which was derived for breast‑conserving surgery only. Extrapolating to mastectomy patients may be inappropriate. Suggest subgroup analysis by T1a/T1b /T2 per TNM staging.**

**Response:** We thank reviewer #2 for this thoughtful observation. Reference 8 originally established 1.4 cm optimal X‑tile cut‑off for patients receiving breast‑conserving surgery. In our manuscript we applied this same cut‑off to the full cohort including mastectomy patients. We fully recognise this limitation.

Given our existing study design: our risk‑stratification model was built from multivariate Cox regression output using this pre‑specified cut‑point; **performing new additional T1a/T1b/T2 stratified survival subgroup analyses would require substantial new re‑running of all PSM‑weighted KM and Cox models beyond current revision timeline, and our primary study objective was to test our predefined five‑variable risk‑stratification scheme.**

Therefore we took two revision actions (Revised manuscript Page 6 and Page 29‑30 Limitations):

1. In Methods‑variable definition section, add explicit note: *“The tumour‑size cut‑off value of 1.4 cm was derived from a prior study focused on breast‑conserving‑surgery patients (reference 8). We applied this same threshold across the whole cohort including mastectomy patients; this represents a potential limitation.”*
2. In Discussion‑Limitations we add text:

“Sixth, our tumour‑size binary cut‑off point of 1.4 cm originates from a prior study limited to breast‑conserving‑surgery patients. We applied this threshold to both mastectomy and breast‑conserving‑surgery participants without separate TNM T‑subgroup stratification (T1a, T1b, T2). Future work should perform separate stratified analyses according to formal AJCC T subcategories for mastectomy versus breast‑conserving‑surgery populations.”

We acknowledge reviewer suggestion as a valuable future research direction, and clearly report this caveat within manuscript.

**Comment 2‑2: Would adding extranodal extension (ENE / ECE) as risk‑factor parameter alter study outcomes?**

**Response**: We appreciate this important suggestion. Unfortunately, **SEER database for breast‑cancer does NOT systematically collect pathological extranodal extension (ENE/ECE) status for our study diagnosis period (2010‑2019)**. This variable is not available for our extracted dataset, so we cannot incorporate ENE/ECE into Cox regression or risk‑stratification models.

We agree ENE is clinically meaningful, but registry‑data constraints prevent us from analysing it in the present work.

**Closing statement**

We believe these revisions have substantially strengthened methodological transparency and addressed all concerns raised by editor and two reviewers. The Mendeley public dataset fulfils PLOS ONE data‑sharing mandate. We are ready to upload three required submission files: Response to Reviewers, Revised Manuscript with Track Changes, clean Manuscript, plus updated high‑resolution Figure 1‑6.

We sincerely appreciate your consideration of our revised manuscript.

Sincerely yours,

Corresponding author: Yewei Yuan

E‑mail: [yuanyewei1268@163.com](mailto:yuanyewei1268@163.com)

Manuscript ID: PONE‑D‑26‑30832
