# Supplementary material for: Effect of different axillary management strategies on survival in elderly patients (≥70 Years) with early-stage breast cancer and 1–2 positive lymph nodes, stratified by risk factors
Source: PLoS One. 2026 Nov 11;21(11):e0353331. doi: 10.1371/journal.pone.0353331 (PMC13754101; doi:10.1371/journal.pone.0353331)
Supplement: Peer review history

# Peer review: Decision Letter 0

**DOI:** 10.1371/journal.pone.0353331.r001

**Authors:** Sanjay Yadav

11 Aug 2026

Dear Dr. chen,

Thank you for submitting your manuscript to PLOS ONE. After careful consideration, we feel that it has merit but does not fully meet PLOS ONE’s publication criteria as it currently stands. Therefore, we invite you to submit a revised version of the manuscript that addresses the points raised during the review process.

Please submit your revised manuscript by Oct 10 2026 11:59PM. If you will need more time than this to complete your revisions, please reply to this message or contact the journal office at plosone@plos.org. When you're ready to submit your revision, log on to https://www.editorialmanager.com/pone/ and select the 'Submissions Needing Revision' folder to locate your manuscript file.

- A letter that responds to each point raised by the academic editor and reviewer(s). You should upload this letter as a separate file labeled 'Response to Reviewers'.
- A marked-up copy of your manuscript that highlights changes made to the original version. You should upload this as a separate file labeled 'Revised Manuscript with Track Changes'.
- An unmarked version of your revised paper without tracked changes. You should upload this as a separate file labeled 'Manuscript'.

If you would like to make changes to your financial disclosure, please include your updated statement in your cover letter. Guidelines for resubmitting your figure files are available below the reviewer comments at the end of this letter.

If applicable, we recommend that you deposit your laboratory protocols in protocols.io to enhance the reproducibility of your results. Protocols.io assigns your protocol its own identifier (DOI) so that it can be cited independently in the future. For instructions see: https://journals.plos.org/plosone/s/submission-guidelines#loc-laboratory-protocols. Additionally, PLOS ONE offers an option for publishing peer-reviewed Lab Protocol articles, which describe protocols hosted on protocols.io. Read more information on sharing protocols at https://plos.org/protocols?utm_medium=editorial-email&utm_source=authorletters&utm_campaign=protocols.

As the corresponding author, your ORCID iD is verified in the submission system and will appear in the published article. PLOS supports the use of ORCID, and we encourage all coauthors to register for an ORCID iD and use it as well. Please encourage your coauthors to verify their ORCID iD within the submission system before final acceptance, as unverified ORCID iDs will not appear in the published article. *Only*  the individual author can complete the verification step; PLOS staff *cannot*  verify ORCID iDs on behalf of authors.

We look forward to receiving your revised manuscript.

Kind regards,

Sanjay Kumar Yadav

Academic Editor

PLOS One

Journal Requirements:

When submitting your revision, we need you to address these additional requirements.

1. Please ensure that your manuscript meets PLOS ONE's style requirements, including those for file naming. The PLOS ONE style templates can be found at

https://journals.plos.org/plosone/s/file?id=wjVg/PLOSOne_formatting_sample_main_body.pdf and

https://journals.plos.org/plosone/s/file?id=ba62/PLOSOne_formatting_sample_title_authors_affiliations.pdf

2. Thank you for stating the following in the Acknowledgments Section of your manuscript:

“Supported by Health Commission of Sichuan Province Medical Science and Technology Program (25LCYJ64)”

We note that you have provided funding information that is not currently declared in your Funding Statement. However, funding information should not appear in the Acknowledgments section or other areas of your manuscript. We will only publish funding information present in the Funding Statement section of the online submission form.

Please remove any funding-related text from the manuscript and let us know how you would like to update your Funding Statement. Currently, your Funding Statement reads as follows:

“Supported by Health Commission of Sichuan Province Medical Science and Technology Program (25LCYJ64)”

Please include your amended statements within your cover letter; we will change the online submission form on your behalf.

3. Thank you for stating the following financial disclosure:

“Supported by Health Commission of Sichuan Province Medical Science and Technology Program (25LCYJ64)”

Please state what role the funders took in the study.  If the funders had no role, please state: "The funders had no role in study design, data collection and analysis, decision to publish, or preparation of the manuscript."

If this statement is not correct you must amend it as needed.

Please include this amended Role of Funder statement in your cover letter; we will change the online submission form on your behalf.

4. Please note that your Data Availability Statement is currently missing the DOI/accession number of each dataset OR a direct link to access each database. If your manuscript is accepted for publication, you will be asked to provide these details on a very short timeline. We therefore suggest that you provide this information now, though we will not hold up the peer review process if you are unable.

5. We note that your Data Availability Statement is currently as follows: “The datasets analyzed in this study were derived from publicly available repositories: SEER database. All raw data and metadata supporting the findings of this study are available within the manuscript and its Supporting Information files.”

Please confirm at this time whether or not your submission contains all raw data required to replicate the results of your study. Authors must share the “minimal data set” for their submission. PLOS defines the minimal data set to consist of the data required to replicate all study findings reported in the article, as well as related metadata and methods (https://journals.plos.org/plosone/s/data-availability#loc-minimal-data-set-definition).

For example, authors should submit the following data:

- The values behind the means, standard deviations and other measures reported;

- The values used to build graphs;

- The points extracted from images for analysis.

Authors do not need to submit their entire data set if only a portion of the data was used in the reported study.

If your submission does not contain these data, please either upload them as Supporting Information files or deposit them to a stable, public repository and provide us with the relevant URLs, DOIs, or accession numbers. For a list of recommended repositories, please see https://journals.plos.org/plosone/s/recommended-repositories.

If there are ethical or legal restrictions on sharing a de-identified data set, please explain them in detail (e.g., data contain potentially sensitive information, data are owned by a third-party organization, etc.) and who has imposed them (e.g., an ethics committee). Please also provide contact information for a data access committee, ethics committee, or other institutional body to which data requests may be sent. If data are owned by a third party, please indicate how others may request data access.

6. Please upload a new copy of Figures 1 - 6 as the detail is not clear. Please follow the link for more information:  https://journals.plos.org/plosone/s/figures

7. If the reviewer comments include a recommendation to cite specific previously published works, please review and evaluate these publications to determine whether they are relevant and should be cited. There is no requirement to cite these works unless the editor has indicated otherwise.

[Note: HTML markup is below. Please do not edit.]

Reviewers' comments:

Reviewer's Responses to Questions

**Comments to the Author**

1. Is the manuscript technically sound, and do the data support the conclusions?

Reviewer #1: Partly

Reviewer #2: Yes

**********

2. Has the statistical analysis been performed appropriately and rigorously?

Reviewer #1: Yes

Reviewer #2: Yes

**********

3. Have the authors made all data underlying the findings in their manuscript fully available?

The PLOS Data policy

Reviewer #1: Yes

Reviewer #2: Yes

**********

4. Is the manuscript presented in an intelligible fashion and written in standard English?

Reviewer #1: Yes

Reviewer #2: Yes

**********

Reviewer #1: This manuscript addresses a clinically important and understudied question, i.e., individualised axillary management in elderly (≥70 years) patients with early-stage breast cancer and 1–2 positive nodes using a well-powered, propensity-matched SEER cohort with a real-world validation component. The study design, risk-stratification approach, and overall conclusions are sound and clinically meaningful. However, certain methodological issues require clarification and revision as have been mentioned in the attachment provided.

Reviewer #2: I would like to inform the authors that the study has been conducted in a very meticulous manner.

However there are some doubts that are of concern.

The tumour size from 1.4 to 5 cm has been taken into consideration despite the fact that TNM staging has very categorical T1b, T1c, T2 defined. Secondly the tumour size with 1.4 to 5 cm is a large range. Though the reference quoted by the authors (ref-8) does categorize the tumour into less than 1.4cm as small tumour, and above 1.4cm is large tumour, the study was purely for BCS. Now extrapolating that to a Mastectomy case would not be a proper. As the indication for radiation therapy in mastectomy of small tumour size was lymph node positivity, as there is no question of BCS in them. So, if there could be a subgroup analysis for according to T1a,T1b, T2 would probably give more insights and a different angle of the study.

Also, Would the extranodal extension (ENE)/ extra capsular extension (ECE) taken into consideration as a parameter in the risk factors make any difference in the results/ outcomes?

**********

what does this mean?). If published, this will include your full peer review and any attached files.

If you choose “no”, your identity will remain anonymous but your review may still be made public.

**Do you want your identity to be public for this peer review?**  For information about this choice, including consent withdrawal, please see our Privacy Policy

Reviewer #1: No

Reviewer #2: **Yes:**  Sasi Mouli

**********

[NOTE: If reviewer comments were submitted as an attachment file, they will be attached to this email and accessible via the submission site. Please log into your account, locate the manuscript record, and check for the action link "View Attachments". If this link does not appear, there are no attachment files.]

To ensure your figures meet our technical requirements, please review our figure guidelines: https://journals.plos.org/plosone/s/figures

You may also use PLOS’s free figure tool, NAAS, to help you prepare publication quality figures: https://journals.plos.org/plosone/s/figures#loc-tools-for-figure-preparation.

NAAS will assess whether your figures meet our technical requirements by comparing each figure against our figure specifications.

Attachment Submitted filename: Reviewer Comments PONE-D-26-30832.docx

# Author comment: Author response to Decision Letter 1

**DOI:** 10.1371/journal.pone.0353331.r002

9 Sep 2026

Manuscript ID: PONE D 26 30832

Manuscript Title: Effect of Different Axillary Management Strategies on Survival in Elderly Patients (≥70 Years) with Early Stage Breast Cancer and 1–2 Positive Lymph Nodes, Stratified by Risk Factors

Dear Academic Editor Dr. Sanjay Kumar Yadav and respected Reviewers,

We sincerely thank you and the reviewers for your thorough evaluation, constructive and insightful comments on our manuscript. We have carefully addressed every comment point by point, revised the manuscript accordingly, and prepared two versions of manuscript files: Revised Manuscript with Track Changes and clean Manuscript. Our raw analysis dataset has been deposited in Mendeley Data (DOI: 10.17632/55brtt8hw9.1) to fulfill PLOS ONE data availability requirements.

Below please find our detailed point by point responses. Page and line numbers refer to the track changed revised manuscript.

Editorial Office Mandatory Requirements

Comment 1. Funding information handling

Thank you for stating the following in the Acknowledgments Section of your manuscript: “Supported by Health Commission of Sichuan Province Medical Science and Technology Program (25LCYJ64)”. Funding information should NOT appear in Acknowledgments section; only fill in online submission form Funding Statement. Please state the role of funders.

Response: We fully understand this PLOS ONE requirement.

1. We have removed all funding related text from the manuscript main body and acknowledgments section (Revised Manuscript, Page 1).

2. Role of funders statement: “The funders had no role in study design, data collection and analysis, decision to publish, or preparation of the manuscript.”

3. We will fill the funding grant number and above funder role statement in the online submission form as instructed.

Comment 2. Data Availability Statement, minimal dataset requirement

Please confirm your submission contains minimal dataset for replication; provide DOI/link for datasets. If restrictions exist, explain.

Response: Our complete minimal dataset supporting all statistical analyses has been publicly deposited to Mendeley Data:

DOI: 10.17632/55brtt8hw9.1 URL: https://data.mendeley.com/datasets/55brtt8hw9/1

We have updated the Data Availability Statement in revised manuscript (Page 2, Methods section):

“All minimal datasets required to replicate findings of this study are publicly available at Mendeley Data, DOI:10.17632/55brtt8hw9.1. The Chinese single center de identified patient dataset is included within the Mendeley Data repository.”

All summary statistics, table values, KM curve source data points are provided in the uploaded repository. No ethical restrictions apply to these de identified data.

Comment3. Figures 1 6 resolution is insufficient, upload new high quality figure files

Please upload new copy of Figures1 6 with sufficient resolution following PLOS ONE figure guidelines.

Response: We have regenerated all six Kaplan Meier survival figures following PLOS ONE illustration specifications (300 DPI, vector friendly format, readable axis labels, legend, tick marks). New figure files will be uploaded during resubmission.

Comment4. Suggest depositing laboratory protocol to protocols.io (optional recommendation)

Response: Thank you for this suggestion. Our analysis workflow is fully described within manuscript Methods section. For this retrospective registry study, we will not deposit a separate protocol.io entry at this stage, but all R/SPSS analysis logic is described in Methods, and raw dataset is available in Mendeley Data for full reproducibility.

Comment5. ORCID for co authors

Encourage all co authors to register and verify ORCID iDs.

Response: We will remind all co authors to complete ORCID registration and verification in Editorial Manager before final acceptance.

Reviewer #1 Comments

Overall comment: This manuscript addresses a clinically important and understudied question, i.e., individualised axillary management in elderly (≥70 years) patients with early stage breast cancer and 1–2 positive nodes using a well powered, propensity matched SEER cohort with a real world validation component. The study design, risk stratification approach, and overall conclusions are sound and clinically meaningful. However, certain methodological issues require clarification and revision.

Comment 1 1: Missing systemic therapy data as major confounder

The manuscript does not report receipt of HER2 targeted therapy or endocrine therapy adherence/duration, both of which are major, well established determinants of breast cancer specific survival (BCSS). Without accounting for systemic therapy exposure, it is difficult to isolate independent contribution of axillary management strategy. Clarify whether these data were available; if unavailable, explicitly acknowledge as significant limitation.

Response: We greatly appreciate this critical comment. The SEER registry database does NOT capture granular information regarding endocrine therapy adherence, duration, or HER2 targeted agent administration for our study period, only simple yes/no chemotherapy flag. This represents an important unmeasured confounder.

We have added explicit discussion of this limitation in Discussion Limitations subsection. Added text excerpt:

“Fourth, SEER database lacks granular systemic therapy information including endocrine therapy adherence/duration and HER2 targeted therapy usage. These variables strongly influence breast cancer specific survival and constitute important unmeasured confounding factors. We could not incorporate these covariates into multivariate Cox models. Therefore, associations observed between axillary treatment strategies and BCSS should be interpreted cautiously. The conclusions are hypothesis generating and require further prospective validation.”

Comment 1 2: Outdated cohort; data extraction lag relative to current clinical practice

SEER data extracted Feb 03 2023; manuscript reviewed ~3 years later. Axillary de escalation practice patterns have evolved. Address generalizability; comment on publication delay; discuss whether newer SEER data could be incorporated.

Response: Thank you for pointing this out. Diagnosis years of our SEER cohort are 2010 2019. Although we performed data pull in Feb 2023, diagnosis time window remains fixed for our study aim. We could not re extract newer SEER diagnosis years (2020 2022): SEER registry requires ~24 months lag for complete survival follow up; patients diagnosed after 2019 lack sufficient 5 year follow up for calculating BCSS and locoregional recurrence endpoints required for our risk stratified analysis. Adding newer cases would render our primary 5 year endpoints unreliable.

We added expanded text in Limitations section :

“Fifth, our SEER study population consists of patients diagnosed between 2010 2019. Although data extraction occurred in February 2023, we selected this diagnosis window to guarantee complete 5 year follow up for BCSS and locoregional recurrence endpoints. More recently diagnosed cases (2020 onwards) in SEER do not yet have mature 5 year survival data. Therefore our findings reflect real world practice from 2010 2019, and contemporary axillary de escalation patterns after 2020 may not be fully represented. Caution should be applied when extrapolating these results to very recent clinical practice.”

Comment 1 3: SLNB / ALND classification is proxy definition (by number of resected nodes), not procedure verified variable

SEER does not record actual axillary surgical intent; we use nodal yield as surrogate. Nodal yield varies independent of operation type, creates misclassification bias. Acknowledge this limitation more directly and discuss potential bias magnitude / direction.

Response: This is a well recognised limitation for SEER based breast cancer axillary surgery studies. Our proxy definition (SLNB = 1 5 lymph nodes removed; ALND ≥ 6 nodes removed) follows widely published prior SEER based breast cancer publications, which we cite in manuscript.

We have strengthened this limitation statement in Limitations (Page 28):

“Third, axillary surgical grouping (SLNB versus ALND) was defined by total number of examined lymph nodes as a proxy measure, since SEER lacks direct procedural codes distinguishing SLNB and ALND. Misclassification bias may exist: rarely, technically extensive SLNB may harvest ≥6 nodes, while incomplete ALND may retrieve fewer than six nodes. Such misclassification could dilute true effect sizes towards null. Nevertheless, this cut off has been repeatedly adopted in peer reviewed SEER breast cancer studies, based on AJCC axillary dissection criteria and published large cohort evidence.”

Comment 1 4: Clarify PSM cohort composition: SEER vs Chinese validation cohort

Methods state “PSM applied to combined cohort”, but table 1 totals correspond only to SEER n=8977. The n=106 Chinese patients are separate unmatched descriptive cohort with no survival analysis. Is the 106 patient cohort included in PSM? If analysed separately, “validation cohort” term may be misleading. Clarify rationale.

Response: Thank you for catching this important wording ambiguity. The 106 Chinese single centre patients were NOT included in propensity score matching. PSM analysis was performed exclusively on the SEER database cohort (n=8977). The Chinese 106 patient dataset serves only as an external descriptive case series, for baseline feature comparison; we did NOT perform survival / recurrence statistical validation on this small sample due to limited event numbers. The original manuscript phrase “combined cohort” was inaccurate wording.

We have revised:

Methods Study Population section (Page 3): removed the misleading wording “combined cohort”. Clearly write: “Propensity score matching was performed exclusively within the SEER database cohort (n = 8 977). An independent Chinese single centre case series (n = 106) was used for descriptive baseline comparison only and was not incorporated into PSM”

The term “validation cohort” has been replaced throughout manuscript with “independent Chinese descriptive case series” to eliminate misinterpretation.

Reviewer #2 Comments

Overall: The study has been conducted in a very meticulous manner. However there are some doubts that are of concern.

Comment 2 1: Tumor size cut off 1.4–5 cm adopted from reference 8 which was derived for breast conserving surgery only. Extrapolating to mastectomy patients may be inappropriate. Suggest subgroup analysis by T1a/T1b /T2 per TNM staging.

Response: We thank reviewer #2 for this thoughtful observation. Reference 8 originally established 1.4 cm optimal X tile cut off for patients receiving breast conserving surgery. In our manuscript we applied this same cut off to the full cohort including mastectomy patients. We fully recognise this limitation.

Given our existing study design: our risk stratification model was built from multivariate Cox regression output using this pre specified cut point; performing new additional T1a/T1b/T2 stratified survival subgroup analyses would require substantial new re running of all PSM weighted KM and Cox models beyond current revision timeline, and our primary study objective was to test our predefined five variable risk stratification scheme.

Therefore we took two revision actions (Revised manuscript Page 6 and Page 29 30 Limitations):

1. In Methods variable definition section, add explicit note: “The tumour size cut off value of 1.4 cm was derived from a prior study focused on breast conserving surgery patients (reference 8). We applied this same threshold across the whole cohort including mastectomy patients; this represents a potential limitation.”

2. In Discussion Limitations we add text:

“Sixth, our tumour size binary cut off point of 1.4 cm originates from a prior study limited to breast conserving surgery patients. We applied this threshold to both mastectomy and breast conserving surgery participants without separate TNM T subgroup stratification (T1a, T1b, T2). Future work should perform separate stratified analyses according to formal AJCC T subcategories for mastectomy versus breast conserving surgery populations.”

We acknowledge reviewer suggestion as a valuable future research direction, and clearly report this caveat within manuscript.

Comment 2 2: Would adding extranodal extension (ENE / ECE) as risk factor parameter alter study outcomes?

Response: We appreciate this important suggestion. Unfortunately, SEER database for breast cancer does NOT systematically collect pathological extranodal extension (ENE/ECE) status for our study diagnosis period (2010 2019). This variable is not available for our extracted dataset, so we cannot incorporate ENE/ECE into Cox regression or risk stratification models.

We agree ENE is clinically meaningful, but registry data constraints prevent us from analysing it in the present work.

Closing statement

We believe these revisions have substantially strengthened methodological transparency and addressed all concerns raised by editor and two reviewers. The Mendeley public dataset fulfils PLOS ONE data sharing mandate. We are ready to upload three required submission files: Response to Reviewers, Revised Manuscript with Track Changes, clean Manuscript, plus updated high resolution Figure 1 6.

We sincerely appreciate your consideration of our revised manuscript.

Sincerely yours,

Corresponding author: Yewei Yuan

E mail: yuanyewei1268@163.com

Manuscript ID: PONE D 26 30832

Attachment Submitted filename: Response to Reviewers.docx

# Peer review: Decision Letter 1

**DOI:** 10.1371/journal.pone.0353331.r003

**Authors:** Sanjay Yadav

27 Oct 2026

Effect of Different Axillary Management Strategies on Survival in Elderly Patients (≥70 Years) with Early-Stage Breast Cancer and 1–2 Positive Lymph Nodes, Stratified by Risk Factors

PONE-D-26-30832R1

Dear Dr. chen,

We’re pleased to inform you that your manuscript has been judged scientifically suitable for publication and will be formally accepted for publication once it meets all outstanding technical requirements.

Within one week, you’ll receive an e-mail detailing the required amendments. When these have been addressed, you’ll receive a formal acceptance letter and your manuscript will be scheduled for publication.

An invoice will be generated when your article is formally accepted. Please note, if your institution has a publishing partnership with PLOS and your article meets the relevant criteria, all or part of your publication costs will be covered. Please make sure your user information is up-to-date by logging into Editorial Manager at Editorial Manager® and clicking the ‘Update My Information' link at the top of the page. For questions related to billing, please contact billing support.

If your institution or institutions have a press office, please notify them about your upcoming paper to help maximize its impact. If they’ll be preparing press materials, please inform our press team as soon as possible -- no later than 48 hours after receiving the formal acceptance. Your manuscript will remain under strict press embargo until 2 pm Eastern Time on the date of publication. For more information, please contact onepress@plos.org.

Kind regards,

Sanjay Kumar Yadav

Academic Editor

PLOS One

Additional Editor Comments (optional):

Reviewers' comments:

Reviewer's Responses to Questions

**Comments to the Author**

Reviewer #1: All comments have been addressed

Reviewer #2: All comments have been addressed

**********

2. Is the manuscript technically sound, and do the data support the conclusions?

Reviewer #1: Yes

Reviewer #2: Partly

**********

3. Has the statistical analysis been performed appropriately and rigorously?

Reviewer #1: Yes

Reviewer #2: Yes

**********

4. Have the authors made all data underlying the findings in their manuscript fully available?

The PLOS Data policy

Reviewer #1: Yes

Reviewer #2: Yes

**********

5. Is the manuscript presented in an intelligible fashion and written in standard English?

Reviewer #1: Yes

Reviewer #2: Yes

**********

Reviewer #1: the authors have addressed all the comments and queires raised in the previous submission upto satisfaction.

Reviewer #2: Ideally it should have been analysed in the way i have asked my first query. Nevertheless kindly try to get the data analysis in the form which i had mentioned previously

**********

what does this mean?). If published, this will include your full peer review and any attached files.

If you choose “no”, your identity will remain anonymous but your review may still be made public.

**Do you want your identity to be public for this peer review?**  For information about this choice, including consent withdrawal, please see our Privacy Policy

Reviewer #1: **Yes:**  Sarrah Idrees

Reviewer #2: **Yes:**  SasiMouli

**********

# Editor report: Acceptance letter

**DOI:** 10.1371/journal.pone.0353331.r004

**Authors:** Sanjay Yadav

PONE-D-26-30832R1

PLOS One

Dear Dr. chen,

I'm pleased to inform you that your manuscript has been deemed suitable for publication in PLOS One. Congratulations! Your manuscript is now being handed over to our production team.

At this stage, our production department will prepare your paper for publication. This includes ensuring the following:

* All references, tables, and figures are properly cited

* All relevant supporting information is included in the manuscript submission,

* There are no issues that prevent the paper from being properly typeset

You will receive further instructions from the production team, including instructions on how to review your proof when it is ready. Please keep in mind that we are working through a large volume of accepted articles, so please give us a few days to review your paper and let you know the next and final steps.

Lastly, if your institution or institutions have a press office, please let them know about your upcoming paper now to help maximize its impact. If they'll be preparing press materials, please inform our press team within the next 48 hours. Your manuscript will remain under strict press embargo until 2 pm Eastern Time on the date of publication. For more information, please contact onepress@plos.org.

You will receive an invoice from PLOS for your publication fee after your manuscript has reached the completed accept phase. If you receive an email requesting payment before acceptance or for any other service, this may be a phishing scheme. Learn how to identify phishing emails and protect your accounts at https://explore.plos.org/phishing.

If we can help with anything else, please email us at customercare@plos.org.

Thank you for submitting your work to PLOS One and supporting open access.

Kind regards,

PLOS One Editorial Office Staff

on behalf of

Dr. Sanjay Kumar Yadav

Academic Editor

PLOS One
